# Isotopic Tracing of Nucleotide Sugar Metabolism in Human Pluripotent Stem Cells

**DOI:** 10.3390/cells12131765

**Published:** 2023-07-03

**Authors:** Federica Conte, Marek J. Noga, Monique van Scherpenzeel, Raisa Veizaj, Rik Scharn, Juda-El Sam, Chiara Palumbo, Frans C. A. van den Brandt, Christian Freund, Eduardo Soares, Huiqing Zhou, Dirk J. Lefeber

**Affiliations:** 1 Department of Neurology, Donders Institute for Brain, Cognition and Behavior, Radboud University Medical Center, 6525 GA Nijmegen, The Netherlands; 2 Department of Clinical Genetics, Maastricht University Medical Center, 6229 HX Maastricht, The Netherlands; 3 Translational Metabolic Laboratory, Department of Laboratory Medicine, Radboud Institute for Molecular Life Sciences, Radboud University Medical Center, 6525 GA Nijmegen, The Netherlands; 4 GlycoMScan B.V., 5349 AB Oss, The Netherlands; 5 hiPSC Hotel, LUMC, 2333 ZC Leiden, The Netherlands; 6 Department of Molecular Developmental Biology, Faculty of Science, Radboud Institute for Molecular Life Sciences, Radboud University, 6525 GA Nijmegen, The Netherlands; 7 Department of Neurology, Amsterdam University Medical Centres, Location Academic Medical Center, Amsterdam Neuroscience, University of Amsterdam, 1098 XH Amsterdam, The Netherlands; 8 Department of Human Genetics, Radboud Institute for Molecular Life Sciences, Radboud University Medical Center, 6525 GA Nijmegen, The Netherlands

**Keywords:** nucleotide sugar metabolism, induced pluripotent stem cells, mass spectrometry-based isotopic tracing, glycosylation, O-GlcNAcylation, PGM1 deficiency

## Abstract

Metabolism not only produces energy necessary for the cell but is also a key regulator of several cellular functions, including pluripotency and self-renewal. Nucleotide sugars (NSs) are activated sugars that link glucose metabolism with cellular functions via protein N-glycosylation and O-GlcNAcylation. Thus, understanding how different metabolic pathways converge in the synthesis of NSs is critical to explore new opportunities for metabolic interference and modulation of stem cell functions. Tracer-based metabolomics is suited for this challenge, however chemically-defined, customizable media for stem cell culture in which nutrients can be replaced with isotopically labeled analogs are scarcely available. Here, we established a customizable flux-conditioned E8 (FC-E8) medium that enables stem cell culture with stable isotopes for metabolic tracing, and a dedicated liquid chromatography mass-spectrometry (LC-MS/MS) method targeting metabolic pathways converging in NS biosynthesis. By ^13^C_6_-glucose feeding, we successfully traced the time-course of carbon incorporation into NSs directly via glucose, and indirectly via other pathways, such as glycolysis and pentose phosphate pathways, in induced pluripotent stem cells (hiPSCs) and embryonic stem cells. Then, we applied these tools to investigate the NS biosynthesis in hiPSC lines from a patient affected by deficiency of phosphoglucomutase 1 (PGM1), an enzyme regulating the synthesis of the two most abundant NSs, UDP-glucose and UDP-galactose.

## 1. Introduction

Besides providing energy and essential metabolites, cellular metabolism is emerging as a key regulator of a plethora of cellular processes in stem cells, including cell growth, proliferation, adhesion, pluripotency, lineage commitment, and differentiation [1,2,3,4,5,6]. Apart from contributing to energy homeostasis, nutrients feed into biosynthesic pathways leading to the formation of metabolic intermediates or end-products that are able to initiate or reinforce pre-established processes in stem cells, for instance by modifying the epigenome, influencing chromatin structure and gene expression, or by modulating post-translational modifications (PTMs) [7,8,9,10,11,12,13,14,15]. Thus, understanding the mechanisms of stem cell metabolism would open novel opportunities for metabolic modulation of cell reprogramming, proliferation, in vitro differentiation, and maturation of stem cells. Yet, the interlinked dynamics by which nutrients flow into alternative metabolic pathways and trigger different cellular responses remain understudied, in part due to challenges posed by application of tracer metabolomics in stem cells [16,17].

**Figure 1:**
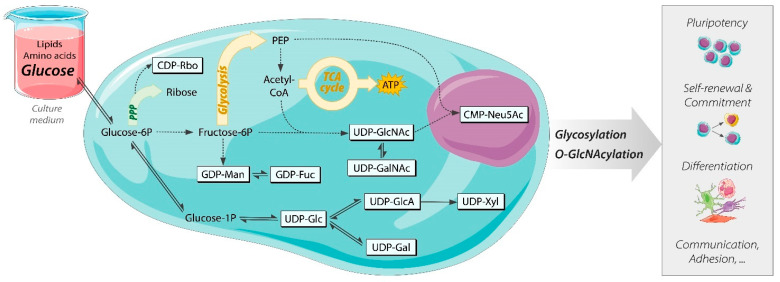
Schematic representation of the nucleotide sugar biosynthesis as metabolic link between glucose and glycosylation, an essential cellular process involved in pluripotency, differentiation, and other stem cell functions. Abbreviations: ATP, adenosine 5′-triphosphate; CDP-Rbo, CDP-ribitol; CMP-Neu5Ac, CMP-N-acetylneuraminic acid; CoA, coenzyme A; GDP-Fuc, GDP-fucose; GDP-Man, GDP-mannose; PPP, pentose phosphate pathway; TCA cycle, tricarboxylic acid cycle; UDP-Gal, UDP-galactose; UDP-Glc, UDP-glucose; UDP-GlcA, UDP-glucuronic acid; UDP-GalNAc, UDP-N-acetylgalactosamine; UDP-GlcNAc, UDP-N-acetylglucosamine; UDP-Xyl, UDP-xylose.

Among the most studied metabolic pathways, glucose metabolism has been reported to be crucial for the metabolic regulation of processes such as self-renewal and differentiation [2,18,19,20] (Figure 1). Glucose is the primary carbon source in human induced pluripotent stem cells (hiPSCs) and embryonic stem cells (hESCs) cultured *in vitro*, being the main substrate of anaerobic respiration via glycolysis and energy storage via glycogen synthesis. Several studies have confirmed that high glycolytic flux is critical for the acquisition and maintenance of pluripotency [18,21,22,23]. Highly active glycolysis in presence of appropriate oxygen concentrations, a phenomenon also known as the Warburg effect, is a typical metabolic occurrence in stem cells, particularly *in vitro*. The preference of anaerobic respiration over aerobic respiration via oxidative phosphorylation is linked to the rapidly proliferating nature of stem cells, which implies a high demand for anabolic precursors for replication, whilst concurrently consuming energy in the form of ATP [18,24]. Furthermore, in vitro glucose represents the main provider of nucleotide sugars, which are activated monosaccharides with a nucleoside mono- or diphosphate moiety attached to the anomeric carbon [25]. Currently, at least ten different nucleotide sugars involved in N-glycosylation and O-GlcNAcylation (Figure 1) have been characterized in human cells, and their biosynthesis involves over 40 different enzymes and multiple metabolic pathways [25,26,27,28].

Glycosylation is an essential PTM consisting of the attachment of oligosaccharides, named glycans, to proteins, which enables the latter to reach their mature and functional configuration. Glycosylation is dependent on the availability of nucleotide sugars. which represent the building blocks of glycan synthesis [15,26,29,30]. Additionally, UDP-*N*-acetylglucosamine (UDP-GlcNAc, Figure 1), a nucleotide sugar produced by the hexosamine biosynthesis pathway (HBP), is also the substrate of O-GlcNAcylation [2,26,31]. The O-GlcNAcylation of several chromatin-rearranging proteins, such as polycomb complexes 1 and 2, and transcriptional factors, such as OCT-4 and SOX2, links the synthesis rate and availability of UDP-GlcNAc with the regulation of essential stem cell processes, such as proliferation and pluripotency maintenance [2,31,32,33,34,35,36,37,38,39]. The HBP is thus considered a metabolic sensor that translates changes in the metabolic pathways into the regulation of cellular processes via UDP-GlcNAc production. The HBP well exemplifies how the synthesis of nucleotide sugar precursors is dependent on multiple converging metabolic branches, including carbohydrate (glucose), nucleotide (uridine triphosphate; UTP), nitrogen (glutamine), and lipid (acetyl-CoA) metabolic pathways (Figure 1) [2,40]. Thus, understanding how these branches contribute to the synthesis of nucleotide sugars (Figure 1) and their fluxes change over time and in response to external stimuli would open new opportunities for metabolic interference to modulate stem cell functions. Nevertheless, these dynamic aspects of the sugar metabolism remain yet to be characterized in stem cells.

Recent advances in mass spectrometry (MS)-based metabolomics enable the investigation of cellular metabolism with an unprecedented level of detail. Yet, simply quantifying the metabolic content of stem cells at a single point in time does not provide information about the reaction rates and their distribution, therefore only offering a static picture of an extremely dynamic and interlinked system [41]. In fact, any changes in enzyme activity caused by metabolic regulation or enzyme deficiency primarily affect enzymatic reaction rates, which may or may not cause changes in the concentrations of metabolites. Metabolic reaction rates and their changes can be inferred from time courses of stable isotopic label incorporation from a labeled substrate (tracer) into intermediates and end products of metabolic pathways via mass spectrometry (MS) [41,42,43,44]. However, as metabolites flow into different metabolic pathways, they become increasingly composite and complex, generating more convoluted isotopic patterns that require increasingly sophisticated analytical approaches and data processing to be interpreted [45].

To enable isotopic tracing experiments in hiPSCs and hESCs, we addressed the need for two key elements. First, we established a chemically defined and customizable version of the E8 culture medium in which glucose and other components (such as glucose, glutamine, and fatty acids) can be replaced with labeled analogs. In parallel, we developed a novel targeted method for liquid chromatography tandem mass-spectrometry (LC-MS/MS) that through careful design of transitions selective for positional labeling in distinct moieties of the nucleotide sugars allows to deconvolute the contribution of individual metabolic pathways converging in nucleotide sugar biosynthesis. Then, we applied our methodology to explore *de novo* nucleotide sugar biosynthesis in hiPSCs, and compared it with other cell types (hESC, fibroblasts) and conditions (healthy vs. patient-derived cells).

## 2. Materials and Methods

### 2.1. Cell Lines and Culture

Two control hiPSC lines (hiPS-1 and hiPS-2), one control hESC line (commercially known as H9 line), and two control fibroblast lines (hFB-1 and hFB-2) were used for the validation of the flux-conditioned E8 (FC-E8) culture medium. Both control hiPSC lines were derived from dermal fibroblasts of healthy donors reprogrammed via polycistronic lentiviral vector according to the protocol reported by Warlich et al. [46] and characterized in Soares et al. [47]. Prior to the experiments, hiPSC and hESC lines were cultured for over 35 passages in Gibco E8 medium (Life Technologies, Carlsbad, CA, USA), according to manufacturer’s instructions. The two primary dermal fibroblast lines were obtained from skin biopsies of healthy donors.

**Figure 2:**
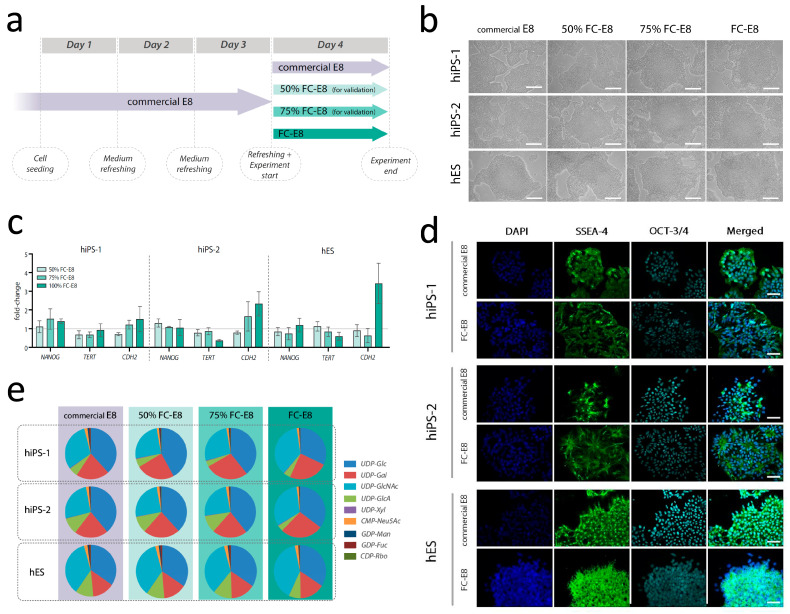
Flux-conditioned E8 (FC-E8) medium maintains pluripotent hiPSC and hESC lines. (**a**) Timeline of the cell culture and the application of FC-E8 medium and mixed media. (**b**) Brightfield pictures of the hiPSC lines and hESC line cultured for 24 h in commercial E8 medium (Gibco), in FC-E8 medium, and in mixed media (50% and 75% FC-E8). Scale bar: 500 µm. A magnified version of these pictures is provided as Supplementary Figure S5. (**c**) Gene expression of pluripotency markers (*Nanog*, *TERT*, and *CDH2*) in two hiPSC lines and a hESC line cultured for 24 h in FC-E8 medium (50%, 75%, or 100%), expressed as fold change compared to the control condition in commercial E8 medium (Gibco, Life Technologies). The data are normalized on the housekeeping gene *ACTG1*. A fold change of 1 indicates equal gene expression between the sample and the control condition (cell cultured in commercial E8). (**d**) Immunofluorescent staining of pluripotency markers (*SSEA-4*, *OCT-3/4*) in hiPSC lines and hESC line cultured for 4 days in commercial E8 medium and in FC-E8 medium. Scale bar: 50 µm. A magnified version of these pictures is provided as Supplementary Figure S7. (**e**) Results of the unlabeled metabolomics analysis on hiPSC lines and hESC line cultured for 24 h in commercial E8 medium, FC-E8 medium, and mixed media (50% FC-E8, 75% FC-E8) (*n* = 4).

For application in a disease model, two hiPSC clones (hiPS_PGM1-1, hiPS_PGM1-2) were generated by lentiviral reprogramming [46] of dermal fibroblasts obtained from a PGM1-deficient patient (c.1507C > T, p.R503X) and pluripotency was verified by immunofluorescent staining (Supplementary Figure S1) and gene expression (Figure 2) of pluripotency markers. Both primary fibroblasts and patient-derived hiPSCs were used under ethical approval protocol 2020-6588 [48,49,50,51,52].

#### 2.1.1. Stem Cell Culture

The hiPSC and hESC lines were cultured at 37 °C with 5% CO_2_ in 6-well plates coated with recombinant human vitronectin (Invitrogen, Thermo Fisher Scientific, Waltham, MA, USA) using commercial E8 medium (Gibco, Life Technologies, Carlsbad, CA, USA), prepared according to manufacturer’s instructions and with the addition of 1% penicillin–streptomycin (10,000 U/mL) (Invitrogen, Thermo Fisher Scientific, Waltham, MA, USA). Every four days, when the cultures reached 65–75% confluence, the cells were detached by incubation for 3–4 min in a solution of 0.5 mM EDTA in 1X Dulbecco’s phosphate-buffered saline (DPBS) without calcium/magnesium (Invitrogen, Thermo Fisher Scientific, Waltham, MA, USA). Next, the EDTA solution was removed and fresh E8 medium was added to suspend the cells, which were then seeded on a new vitronectin-coated 6-well plate with densities of 150 × 10^3^ cells/well, or in a 12-well plate with density 20 × 10^3^ cells/well for immunofluorescence staining. The plates were placed in a 37 °C incubator with 5% CO_2_, and the commercial E8 culture medium was refreshed every 24 h (Figure 2a).

For the medium validation experiments, FC-E8 medium was mixed in incremental concentrations with commercial E8 medium to test the survival and proliferation of the cell lines and their adaptation at different levels of FC-E8 medium. Four conditions were tested: (i) commercial E8 medium, (ii) 100% FC-E8 medium, (iii) 50% E8 medium mixed with 50% FC-E8 medium, and (iv) 25% E8 medium mixed with 75% FC-E8 medium. The cells were refreshed with FC-E8 medium or mixed media (50% FC-E8; 75% FC-E8) after 72 h from seeding and incubated up to 24 h (Figure 2a).

#### 2.1.2. Primary Fibroblast Culture

Primary dermal fibroblasts were cultured in DMEM containing 5.5 mM glucose (Gibco, Life Technologies, Carlsbad, CA, USA), and supplemented with dialyzed 10% fetal bovine serum (FBS) (Gibco, Life Technologies, Carlsbad, CA, USA) and 1% penicillin/streptomycin 10,000 U/mL (Gibco, Life Technologies, Carlsbad, CA, USA). Cells were seeded with density 1.5 × 10^6^ in T75 flasks and incubated at 37 °C and 5% CO_2_. Upon reaching 80% confluence (~72 h), the fibroblasts were washed with 5 mL of 1X PBS, and then incubated with 1 mL of 1:250 trypsin in 1X PBS solution (Life Technologies, Carlsbad, CA, USA) for up to 10 min at 37 °C, regularly checking the cell detachment via optic microscope. Once the cells were detached, trypsin was neutralized by addition of 9 mL of medium, and the resulting cell-containing solution was seeded in new flasks. For our experiments, we used primary fibroblast lines below passage 25.

### 2.2. Preparation of the Flux-Conditioned E8 (FC-E8) Medium

To perform tracer metabolomics on hiPSCs and hESCs, a customizable, chemically defined medium, named flux-conditioned E8 (FC-E8) medium, was prepared based on the formulation of the Essential 8 (E8) medium described by Chen et al. [53] for stem cell culturing, but with some changes, such as the amount of sodium carbonate and the absence of HEPES (Supplementary Table S1). In brief, custom DMEM/F-12 powder (Gibco, Life Technologies) was dissolved in ultrapure Versol water (Laboratoire Aguettant, Champagne, FR, USA), after which several compounds were manually added according to the formulation reported in Supplementary Table S1. Besides the formulation, the pH and osmolality values were also changed from Chen et al. [53] and optimized to ensure the viability and pluripotency of our lines. The medium osmolality was tested with an Osmometer Mod. 3320 (Advanced Instruments, Inc., Norwood, MA, USA) calibrated with 50 and 850 mOsm/kg standard solutions (double-point calibration), and it was adjusted to 308 (±10) mOsm/kg using ultrapure water. The medium pH was adjusted to 7.3 (±0.05) by addition of 1 M HCl prior to application. A light version of FC-E8 medium was prepared by addition of unlabeled glucose (Sigma-Aldrich, St. Louis, MO, USA) to a concentration of 17.5 mM for validation experiments and unlabeled metabolomics experiments, while a heavy version was prepared by addition of ^13^C_6_-glucose (Omicron Biochemicals, Inc., South Bend, IN, USA) to a concentration of 17.5 mM.

### 2.3. Phosphoglucomutase Enzymatic Activity Assay

Phosphoglucomutase activity was measured in cell lysates from control hiPSCs (hiPS-1, hiPS-2) and from patient-derived PGM1-deficient hiPSCs (hiPS_PGM1-1, hiPS_PGM1-2) using a Konelab 20XTi (Thermo Fisher Scientific, Waltham, MA, USA). Cells were washed with 1X PBS (Gibco, Life Technologies, Carlsbad, CA, USA), detached with 0.5 mM EDTA solution (Invitrogen, Thermo Fisher Scientific, Waltham, MA, USA), and pelleted via centrifugation at 550× *g* for 5 min at room temperature. Next, the pellets were washed with 6 mL of 0.9% NaCl three times, and afterwards the dry pellets were snap-frozen in liquid nitrogen and stored at −80 °C till measurement. The enzyme activity assay performed was based on the quantification of NADPH production, measured by absorbance at 340 nm according to Van Schaftingen and Jaeken [54]. Data on PGM activity were normalized on protein content, also measured using a Konelab 20XTi. Another enzyme, namely phosphomannose isomerase (PMI), was used as control. Data analysis and visualization were performed in Microsoft Office Excel and PRISM GraphPad 5.03.

### 2.4. Gene Expression Analysis of Pluripotency Markers

hiPSCs and hESCs were seeded and cultured in commercial E8 medium for 72 h, followed by replacement with (light) FC-E8 medium. RNA was extracted 24 h after the medium replacement, when the culture confluence reached 60–70%. Cells were lysed by addition of 1 mL of TRIzol reagent per well (Ambion, Austin, TX, USA), and the lysates were snap-frozen in liquid nitrogen and stored at −80 °C until extraction. RNA extraction was performed with Direct-zol RNA MiniPrep Plus kit (Zymo Research, Irvine, CA, USA), according to manufacturer’s instructions. After extraction, RNA extracts were used for cDNA synthesis performed with RevertAid First Strand cDNA Synthesis Kit (Thermo Fisher Scientific, Waltham, MA, USA), according to manufacturer’s specifications. cDNA samples were diluted 10× with nuclease-free water (Promega, Madison, WI, USA) and used for expression analysis via quantitative real-time PCR (qRT-PCR) of three pluripotency markers: *Nanog*, *CDH2*, and *TERT*. The *ACTG1* gene was chosen as housekeeping gene for comparison of gene expression and normalization. Primer sequences were generated via Primer-Blast (ncbi.nlm.nih.gov/tools/primer-blast/ (accessed on 5 January 2023)) (Supplementary Table S2), and the primers were optimized and validated via qRT-PCR to exclude the presence of off-targets prior to application. qRT-PCR was performed in triplicate using the GoTaq PCR Master Mix (Promega, Madison, WI, USA) according to manufacturer’s specifications. A CFX96 Real-Time PCR thermocycler and detection system (BioRad, Hercules, CA, USA) was used for the quantification of SYBR Green (Promega, Madison, WI, USA) signal. The C_t_ values were recorded using CFX manager software (version 2.0; BioRad, Hercules, CA, USA) and used to derive the expression fold-change using the 2^−ΔΔCt^ method [55]. Data analysis and visualization were performed in Microsoft Office Excel and PRISM GraphPad (version 5.03).

### 2.5. Immunofluorescent Staining of Pluripotency Markers

To perform immunofluorescent staining, hiSPCs and hESCs were refreshed in light FC-E8 medium, and after 24 h they were passaged and seeded in a 12-well plate containing borosilicate cover-glasses, and further culture for 4 days in light FC-E8 medium. Once ready, the cells were washed twice with DPBS 1X without calcium/magnesium (Gibco, Life Technologies, Carlsbad, CA, USA), and then incubated for 15 min at room temperature with 700 μL per well of 4% PFA as fixative solution (Sigma-Aldrich, St. Louis, MO, USA). The fixed cells were washed three times with 1 mL/well of PBS 1X (Roche, Basel, CH, USA) (5 min/wash), and the cover-glasses were then preserved in 700 μL per well of glycerol 50% (Sigma-Aldrich, St. Louis, MO, USA) at 4 °C until staining. For staining, the glycerol solution was removed by six washing steps each using 1 mL/well of PBS 1X (30 s/wash). Next, the cells were permeabilized by addition of 1 mL/well of 0.1% Triton X-100 in 3% BSA/1X PBS solution, and incubated for 10 min at 4 °C. The coverslips were then washed three time with PBS 1X (5 min/wash), as performed before permeabilization, and then incubated with 1 mL/well of 3% BSA/1X PBS (blocking solution) for 30 min at room temperature. After three more washes in PBS 1X, cells were incubated with the primary antibodies against OCT-3/4 (nuclear marker) and SSEA-4 (membrane marker) for 1 h at room temperature in a wet chamber. The anti-human OCT-3/4 mouse IgG2b antibody (Santa Cruz Biotechnology Inc., Dallas, TX, USA) was diluted 1:70, while the anti-human SSEA-4 mouse IgG3 (BioLegend, San Diego, CA, USA) was diluted 1:100. Next, the cover-glasses were washed three times in 100 uL 0.05% Tween-20 in 1X PBS (10 min/wash), and then incubated with the secondary fluorochrome-conjugated antibodies Alexa 647 goat anti-mouse IgG2b (Invitrogen, Thermo Fisher Scientific, Waltham, MA, USA) and Alexa 488 goat anti-mouse IgG3, Alexa 488 (Invitrogen, Thermo Fisher Scientific, Waltham, MA, USA), both diluted 1:350, for 1 h at room temperature in the dark. The over-glasses were then washed again three times in 100 μL 0.05% Tween-20 in 1X PBS, and then incubated with 100 μL of 0.75% DAPI solution in PBS 1X (Molecular Probes, Eugene, OR, USA) for 6–7 min in the dark. Lastly, the cover-glasses were dipped in Versol ultra-pure water (Laboratoire Aguettant, Champagne, FR, USA) and mounted on a microscope slide using ProLong Diamond Antifade Mountant (Molecular Probes, Eugene, OR, USA). The following day, pictures were taken using a LEICA DMI6000B widefield fluorescence microscope (Leica Microsystems, Wetzlar, DE, USA).

### 2.6. Metabolomics Experiments

#### 2.6.1. Unlabeled Metabolomics Experiments in Stem Cells

Both hiPSC lines and hESC line were grown on vitronectin-coated 6-well plates until ~70% confluence. Then, the cells were refreshed with the light version of FC-E8 medium and incubated at 5% CO_2_ and 37 °C for 24 h before quenching [28].

#### 2.6.2. Isotopic Labeling Experiments in Stem Cells

hiPSC and hESC lines were seeded in 6-well plates in commercial E8 medium (Gibco, Life Technologies, Carlsbad, CA, USA) at 0.15 × 10^6^ cells/well in six replicates per intended timepoint (15 s; 5, 10, 15, 25, 45, 75 min; 4, 10, 24 h). After 24 h the culture medium was replaced by heavy FC-E8 medium containing ^13^C_6_-glucose, with the exception of timepoint 0 that was quenched without medium switch and used to obtain information about the status of the cells at the start of the labeling experiment. For the initial timepoint heavy FC-E8 medium was added to all wells and removed immediately after, and this procedure accounted for a total time of 15 s (timepoint 0).

#### 2.6.3. Isotopic Labeling Experiments in Primary Dermal Fibroblasts

Fibroblasts were grown and maintained in M-199 medium (Lonza, Basel, CH, USA) supplemented with 10% FBS (Gibco, Life Technologies, Carlsbad, CA, USA) and 1% penicillin/streptomycin 10,000 U/mL (Gibco, Life Technologies, Carlsbad, CA, USA). Cells were seeded at 0.15 × 10^6^ cells/well in 2 mL DMEM 5 mM glucose (Gibco, Life Technologies, Carlsbad, CA, USA), 10% dialyzed FBS per well on a 6-well plate in triplicate. After 2 days, cells were refreshed with heavy DMEM supplemented with 5 mM ^13^C_6_-glucose and 10% dialyzed FBS and quenched per intended timepoint (0, 10 min; 1, 4, 7, 24 h). Plates for time point 24 h were seeded using 0.1 × 10^6^ cells to compensate for growth during labeling. Plates were incubated for 72 h at 37 °C and 5% CO_2_ before switching to heavy DMEM at timepoint 0 of the experiment. The timepoint 0 plate was not exposed to labeled medium.

#### 2.6.4. Quenching of Metabolism

To quench the cells prior to metabolite extraction, the plates were washed twice with 2 mL/well of 75 mM ammonium carbonate solution (Fluka, Honeywell, Charlotte, NC, USA) adjusted to pH 7.3 (±0.05) with acetic acid. After removing the washing solution, the plates were snap-frozen by contact of the underside of the plate with liquid nitrogen and stored at −80 °C until metabolite extraction of metabolites, as described in van Scherpenzeel et al. [28]. The same quenching procedure was used for both the unlabeled experiments and the isotopic labeling experiments.

#### 2.6.5. Polar Metabolite Extraction from Adherent Cells

Polar metabolites were extracted using a −20 °C cold extraction solution of 40:40:20 *v*/*v* acetonitrile (Biosolve, Dieuze, FR, USA), methanol (Honeywell, Charlotte, NC, USA), and ultra-pure Versol water (Laboratoire Agguetant, Champagne, FR, USA). While keeping the plates on ice, each well was incubated for five minutes with 1.4 mL of extraction solution, while gently rocking the plate to ensure homogeneous permeabilization. Next, the extracts were centrifuged at 13,000 rpm for 3 min at 4 °C to pellet and remove cell debris. Lastly, the supernatants were transferred to a separate tube, and dried overnight in a vacuum using a Savant SC100 SpeedVac concentrator (RVT 100) equipped with an external oil pump (Spectralab Scientific Inc., Markham, ON, USA). The extracts were stored at −80 °C and dissolved in 100 μL ultra-pure Versol water prior to analysis.

#### 2.6.6. Nucleotide Sugar Analysis by Ion-Pairing UHPLC-MS/MS

The analytes were separated by ion-pairing ultra-high performance liquid chromatography (UHPLC) via an Agilent 1290 Infinity UHPLC system (Agilent, Santa Clara, CA, USA) equipped with an Acquity HSS T3 column (Waters, Milford, MA, USA) using the method, buffers, and separation gradient as described by van Scherpenzeel et al. [28,56]. The MS analysis was performed on an Agilent 6490A QqQ UHLC-MS/MS, equipped with high-flow iFunnel ionization source, and controlled by Agilent MassHunter Workstation Software (version B.08.02). The Agilent Jet Stream ion source was operated in negative ion mode at 3500 V capillary voltage, 2000 V nozzle voltage, sheath gas flow of 12 L/min (nitrogen) at 200 °C temperature, drying gas flow of 15 L/min at 200 °C temperature, nebulizer gas flow at 20 psi, iFunnel high-pressure RF 110, and low-pressure RF 100. Data acquisition was performed in multiple-reaction monitoring (MRM) as reported by van Scherpenzeel et al. [28]. Q1 and Q3 were set to unit resolution (0.7 FWHM) and 10 ms dwell time [28]. Q1 and Q3 set to unit resolution (0.7 FWHM), 10 ms dwell time, and 4 V cell accelerator potential were used for all MRM ion pairs.

A mix of commercial standards for eight nucleotide sugars, plus CDP-ribitol (synthesized in-house according to van Tol et al. [57]), was used to confirm the identity of analyzed compounds and as quality check for the sample (Supplementary Table S3). After acquisition, peak integration was performed using Skyline (version 20.1), while data analysis and visualization were performed in Microsoft Office Excel and PRISM GraphPad (version 5.03). Due to insufficient chromatographic separation of UDP-GlcNAc and UDP-GlcNAc, total signal of both isomers was integrated as UDP-GlcNAc. Based on peak shape and MRM transitions the ratio between these isomers remained constant in all analyzed samples.

#### 2.6.7. MS Data Processing and Visualization

For unlabeled experiments, after integration, the peak areas were normalized to the sum of all nucleotide sugars detected (total peak area) to obtain relative abundances.

**Figure 3:**
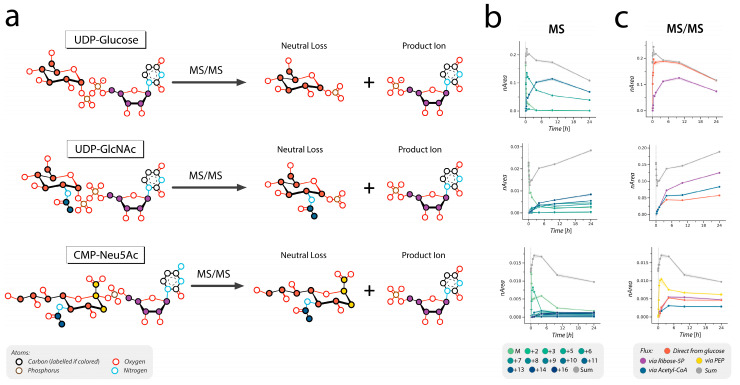
Deconvolution of carbon fluxes contributing to UDP-Glc, UDP-GlcNAc, and CMP-Neu5Ac synthesis. (**a**) Schematic representation of the structures of the nucleotide sugars and products of their fragmentation, including positions of labels derived from different metabolic precursors. Fragmentation of the nucleotide sugar molecule along the phosphodiester bond allows for independent detection of labeled variants of the sugar moiety (neutral loss) and the nucleotide moiety (product ion) as shown for three nucleotide sugars in this figure (and for the other nucleotide sugars of interest in Supplementary Figure S2). (**b**,**c**) Time courses of isotopologues separated only based on their precursor mass (**b**), and after label deconvolution based on MS/MS data (**c**).

**Figure 4:**
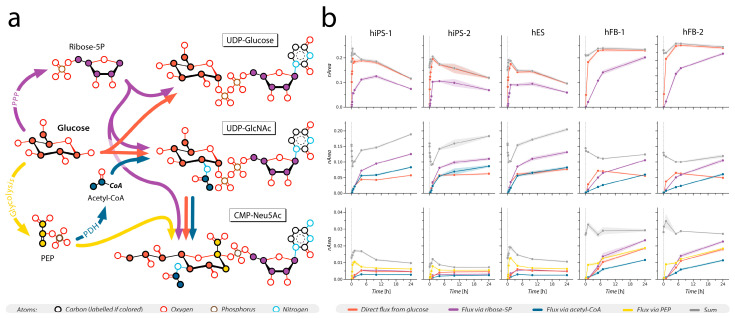
Incorporation of ^13^C atoms from ^13^C_6_-glucose into nucleotide sugars in hiPSCs mirrors the dynamics observed in hESCs but not in primary fibroblasts. (**a**) Schematic flux of ^13^C atoms from glucose to three major nucleotide sugars, UDP-glucose, UDP-*N*-acetylglucosamine (UDP-GlcNAc), and CMP-*N*-acetylneuraminic acid (CMP-Neu5Ac). (**b**) Results of the isotopic labeling experiments show the time course of incorporation of the labeled atoms in UDP-glucose, UDP-GlcNAc, and CMP-Neu5Ac in two hiPSC lines (hiPS-1, hiPS-2), in one hESC line (hES), and in two primary dermal fibroblast lines (hFB-1, hFB2) (*n* = 6 for stem cells, *n* = 3 for fibroblasts). *Y*-axis: normalized area (nArea) based on the total peak area. *X*-axis: time expressed in hours.

**Figure 5:**
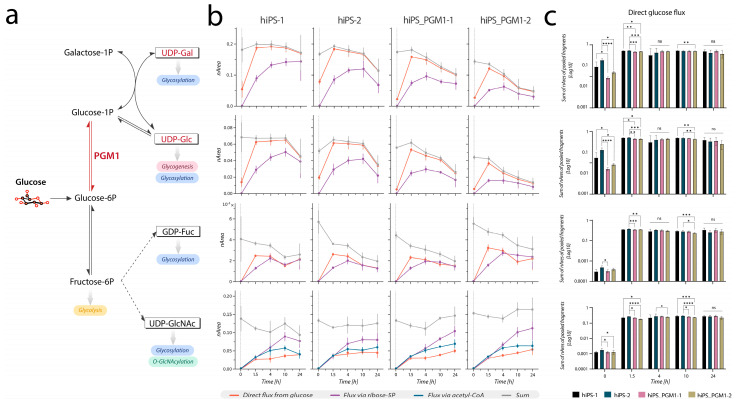
Time course of metabolic labeling of nucleotide sugars in control hiPSC lines versus PGM1-deficient hiPSC lines. (**a**) Scheme of the metabolic pathways affected by phosphoglucomutase (PGM1) deficiency. PGM1 (indicated in red) mediates the interconversion of glucose 6-phosphate and glucose 1-phosphate. Legend: double arrows indicate bidirectional reactions, solid arrows indicate direct reactions, dashed arrows indicate a series of multiple reactions (omitted in the figure). (**b**) Time-course of ^13^C incorporation in UDP-Glc, UDP-Gal, GDP-Fuc, and UDP-GlcNAc. Other nucleotide sugars are shown in Supplementary Figure S4. (**c**) Bar plots of the sums of the normalized intensities of the collisional fragments deriving from the ‘direct flux via glucose’ (orange line in panel **b**). The significance of the differences among control lines versus patient-derived lines was evaluated via Kruskal–Wallis test (only significant differences are shown: * *p* < 0.05, ** *p* < 0.01, *** *p* < 0.001, **** *p* < 0.0001, ns non significant; *n* = 6).

For isotopic labeling experiments, the acquisition method was extended to include additional transitions that cover expected labeled variants of precursor and product ions. The MS was operated in dynamic MRM mode with an updated transitions list to account for isotope incorporation (Supplementary Table S4). Integrated peak areas were normalized to the sum of all isotopologues of each nucleotide sugar. Transitions selective for positional labeling in distinct moieties of nucleotide sugars were grouped and summed to obtain total signal corresponding to isotopomers labeled through a specific biosynthetic pathway (Supplementary Table S5). Deconvoluted pathways include flux directly from glucose (orange in Figure 3, Figure 4 and Figure 5 and Supplementary Figures S2–S4), flux via ribose-5-phosphate (purple), via acetyl-CoA (blue), and via PEP (yellow). The sum of signals for all isotopomers was calculated to obtain the total signal per molecule (grey in Figure 3, Figure 4 and Figure 5 and Supplementary Figures S2–S4).

To evaluate the significance of the difference in the direct glucose flux among the control hiPSC lines (hiPS-1, hiPS-2) versus the patient-derived hiPSC lines (hiPS_PGM1-1, hiPS_PGM1-2), we proceeded as follows. First, we summed the (normalized) areas of all the product ions and neutral loss fragments deriving from the direct flux via glucose (Figure 5b) and, after log_10_-transformation, the significance was evaluated via non-parametric Kruskal–Wallis test (with uncorrected Dunn’s test, α = 0.05) (Figure 5c).

## 3. Results

### 3.1. Flux-Conditioned E8 (FC-E8) Medium Allows the Use of Labeled Metabolic Tracers in Human Pluripotent Stem Cells

We aimed to establish a culture medium that enables metabolite tracing of multiple key metabolic pathways, by replacing medium components with isotopically labeled analogs (Supplementary Table S1). Incubation of hiPSCs and hESCs with FC-E8 medium was tested for up to four days, during which the cells remained pluripotent and comparable with the cells cultured in commercial E8 medium (Figure 1). Morphological examination via optic microscopy revealed compacted, monolayer colonies resembling the cells cultured in commercial E8 for both hiPSC and hESC lines, with only slight elongation of the cells at the edges of the colonies in the hiPS-2 line.

To confirm pluripotency, gene expression levels of pluripotency markers *Nanog*, *TERT*, and *CDH2* were analyzed. Levels were comparable across culture conditions in the hiPS-1, hiPS-2, and hESC lines and comparable with culture in commercial E8, although *CDH2* expression was increased in hESC in full FC-E8 medium (Figure 2c). Immunofluorescence staining of SSEA-4 and OCT-3/4 pluripotency markers after expansion and culture in FC-E8 medium for four days confirmed pluripotency (Figure 2d). Rosette-like formation was observed in hiPS-2, which also showed an increased *CDH2* expression (Figure 2c,d). Analysis of cell extracts collected after 24 h incubation in different culture media showed comparable relative levels of nucleotide sugars in the hiPS-1, hiPS-2, and hES lines, with no clear differences that could be related to medium switch (Figure 2e). Together, these data indicate that the FC-E8 medium is able to maintain the expression of pluripotency markers in both hiPSCs and hESCs for a window of time sufficient for isotopic tracing of metabolic pathways.

### 3.2. Time Course of Isotopic Tracing into Nucleotide Sugars in Pluripotent Stem Cells

Isotopic tracing of ^13^C_6_-glucose into nucleotide sugars integrates multiple metabolic pathways. By determining the positional labeling of carbons in the product ions of each nucleotide sugar molecule, we could identify the enzymatic reaction from which those labeled carbons are derived and thus determine the time course of incorporation via that metabolic pathway, ultimately deconvoluting independent metabolic fluxes (Figure 3). These include direct incorporation of labeled carbons from glucose into the hexose ring of the sugar moiety of the nucleotide sugar (Figure 3, in orange), and the incorporation through the pentose phosphate pathway (PPP) into the ribose ring of the nucleotide moiety (Figure 3, in purple). For UDP-GlcNAc and CMP-Neu5Ac, two additional metabolic pathways converge onto their biosynthesis, including a PDH-linked shunt from glycolysis, which provides two ^13^C atoms for the *N*-acetyl group (Figure 3, in blue) and glycolysis-derived phosphoenolpyruvate (PEP) (Figure 3, in yellow), which provides three ^13^C atoms to the 9-carbon containing sialic acid core structure.

This isotopic tracing method was applied to the analysis of nucleotide sugar biosynthesis in pluripotent stem cells (hiPSCs and hESCs) and compared to primary dermal fibroblasts, which represent the cell type of origin of the two control hiPS lines (Figure 4). Overall, the time courses of label incorporation observed in hiPS-1 and hiP-2 mirror the labeling in hES, whilst differing from the time courses in control primary fibroblasts (Figure 4b, Supplementary Figure S3). By summing signals from all detected isotopomers, the evolution of the total pool of each metabolite could be evaluated, again showing striking differences between the stem cell lines and primary fibroblasts (Figure 4b, in grey). In UDP-Glc for instance, the rate of incorporation of labeled carbons flowing directly from glucose (Figure 4c, in orange) or through the PPP via ribose 5-phosphate (Figure 4c, in purple) is higher in stem cells, reaching the maximum labeling fraction already at 1.5 h, while in primary fibroblasts it appears delayed, as it reaches the maximum around 4 h for direct flux and more than 24 h for flux via ribose 5-phosphate. Likewise, the dynamics of the carbon flux in glycolysis via pyruvate dehydrogenase (PDH) to acetyl-CoA, which contributes the acetyl group in UDP-GlcNAc, is also faster than in primary fibroblasts. Lastly, the carbon flux flowing through glycolysis and phosphoenolpyruvate (PEP), used for synthesis of CMP-Neu5Ac, reaches the maximum peak at 1.5 h in all three stem cell lines, while in fibroblasts the maximum label incorporation has not yet been reached at 24 h.

To summarize, we resolved (i) which fluxes converged into each nucleotide sugar in hiPSCs, and (ii) showcased different time courses of label incorporation from each metabolic flux between different nucleotide sugars. (iii) Lastly, our data showed how the flux dynamics in hiPSCs mirrors hESCs, as opposed to dermal fibroblasts.

### 3.3. Metabolic Tracing in PGM1-Deficient hiPSCs Shows Reduced UDP-Glc and UDP-Gal Pool Sizes and Mildly Delayed Initial Synthesis Rates

Next, we used the developed technology to study perturbances in nucleotide sugar biosynthesis in phosphoglucomutase 1 (PGM1) deficiency (MIM#614921), a congenital disorder of glycosylation (CDG) affecting the synthesis of the nucleotide sugars UDP-Glc and UDP-Gal (Figure 4a).

First, phosphoglucomutase activity was measured in all four hiPSC lines, showing a residual activity in both patient-derived lines of less than 4% compared to the controls (Table 1), thus confirming the enzymatic deficiency. Unlabeled metabolomics analysis in PGM1-deficienct hiPSC lines (hiPS_PGM1-1 and hiPSC_PGM1-2) showed a reduced pool of UDP-Glc and UDP-Gal (Supplementary Figure S7), consistent with previous reports on PGM1 deficient fibroblasts [49]. Additionally, an increased pool of UDP-GlcA and UDP-GlcNAc was observed (Supplementary Figure S7). Next, we investigated the time courses of ^13^C carbon incorporation through the biosynthesis of these nucleotide sugars. As expected for cells harboring mutations in *PGM1*, we observed significant initial delay in the direct flux from glucose to UDP-Glc and UDP-Gal, whose synthesis is dependent on this enzyme (Figure 5b, in orange, and Figure 5c, first two timepoints). As the patient-derived hiPSCs reached the same isotopic steady-state as the controls, no relevant difference in the label incorporation rate was observed after 1.5 h. Label incorporation through the PPP and ribose 5-phosphate showed initial no delay (Figure 5, in purple). Furthermore, no clear delay at the initial timepoints was observed for the other nucleotide sugars analyzed, as expected since their synthesis does not directly depends on PGM1 enzymatic reaction (Figure 5b,c, Supplementary Figure S4). Notably, total amounts of UDP-Glc and UDP-Gal (Figure 5b) differed between controls and PGM1-deficient lines, showing a decrease in both metabolites in PGM1-deficient lines. As a result, the levels of these nucleotide sugars, which were similar at timepoint 0, decreased at timepoint 24 h, consistently with results of the equivalent experiment performed without labeled glucose (Supplementary Figure S4).

## 4. Discussion

In recent years it has become increasingly clear that cellular metabolism is a key regulator of several cellular functions in pluripotent stem cells, including proliferation, pluripotency maintenance, lineage specification, and commitment [58,59,60]. An emblematic example of this is represented by nucleotide sugar biosynthesis, which directly links carbohydrate metabolism with many specialized stem cell functions, mostly via glycoconjugates generated through N-glycosylation and O-GlcNAcylation [25,26,27,28]. Hence, understanding how different metabolic pathways converge onto the synthesis of nucleotide sugars, and how these metabolites change over time and in response to external stimuli could offer new opportunities for metabolic modulation of stem cell functions. However, this implies the study of a highly dynamic and interlinked system, which cannot be satisfied by metabolite quantification alone. Stable isotope tracing by mass spectrometry (a.k.a. tracer-based metabolomics) offers the possibility of studying the flow of individual atoms through metabolic transformations by detecting the incorporation of stable isotope within metabolites to infer or estimate metabolic reaction rates and their distribution [41,42,43,44]. However, its application still remains limited in stem cell models, largely due to a lack of chemically defined and customizable culture media allowing replacement of nutrients with labeled analogs [17].

In this study, we successfully developed the tools to enable isotopic tracing in human (pluripotent) stem cells to explore de novo nucleotide sugar biosynthesis. First, we established a chemically defined and customizable version of the E8 culture medium, namely, FC-E8, in which glucose could be replaced with its labeled analogs for stable isotope tracing while maintaining the conditions to ensure cell pluripotency for at least a 24-h window (Figure 2). However, the increased *CHD2* expression in hES and hiPS-2, along with the apparent formation of rosette-like structures in at latter after prolonged culture in FC-E8 medium (Figure 2d), should be carefully considered in the case of application of this medium in flux studies longer than 24 h.

In parallel, we optimized a targeted ion-paring UPHLC-MS/MS method for detection of labeled isotopologs of nucleotide sugars and identification of positional labeling (Figure 3), to derive the time course of carbon incorporation via different metabolic pathways converging in nucleotide sugar synthesis [61,62,63,64].

After validation of our culture medium and targeted MS method, we detected up to nine nucleotide sugars in hiPSCs (Figure 1), whose pool sizes strikingly resembled those in hESCs, but greatly differed from the levels detected in fibroblasts, representing the cell type used for reprogramming (Figure 4, Supplementary Figure S3). Moreover, metabolic tracing showed that the dynamics of labeled carbon incorporation in nucleotide sugars detected in both hiPSC lines closely mirrored hESCs but differed from the incorporation time course detected in dermal fibroblasts (Figure 4, Supplementary Figure S3). Previous studies proposed the existence of a somatic metabolic memory, stemming from the concepts of epigenomic and transcriptomic memory of hiPSCs [1,65]. Several studies have postulated that hiPSCs tend to maintain an epigenomic and transcriptomic profile reminiscent of the cells of origin, such as dermal fibroblasts, despite their pluripotent state and resemblance to hESCs [65,66,67,68]. Recently, speculations about the existence of a somatic metabolic memory have also been raised [1,69,70], although no fully conclusive evidence has been produced yet. Regarding the nucleotide sugar biosynthesis, our data do not confirm the presence of a metabolic memory since the pathway greatly differs between hiPSCs and fibroblasts both by metabolite abundances and time course of the synthesis.

Next, we tested our protocol for isotopic tracing in hiPSCs derived from a patient with a genetic defect affecting the nucleotide sugar synthesis, namely, PGM1 deficiency. PGM1 deficiency is a rare congenital metabolic disorder in which the enzyme responsible for the interconversion of glucose 6-phosohate and glucose 1-phosphate is affected by loss of function [49]. *PGM1* mutations result in reduced pools of UDP-Glc and UDP-Gal [49,52]. Our findings confirmed reduction of UDP-Glc and UDP-Gal pool sizes, although less dramatic than previously reported in primary patient-derived fibroblasts [52], and their faster decrease over time as result of increasing glucose depletion in the culture medium (Figure 5). However, only an initial delay in label incorporation rate though the affected branch of the synthetic pathway was detected in hiPSCs (Figure 5). It remains to be determined if PMG1 enzyme maintains excess enzymatic capacity within hiPSCs under the conditions studied. However, we could speculate that the modest difference in the observed incorporation rates compared to the results of the enzymatic assay may suggest that the flux via PGM1 required in cell culture is far below V_max_. Additionally, other PGM isoforms, such as PGM2, might partially compensate for the deficiency by contributing to the catalysis of the affected reaction.

Our data also show how culture conditions and time of harvesting after medium refreshing may have a critical influence on the outcome of cellular metabolomics experiments, especially when using hiPSCs to model metabolic disorders. A careful assessment of the culture conditions, starting with (but not limited to) the medium formulation, can help in minimizing potential artifacts and the risks of concealing or enhancing pathogenic effects caused by the genetic defects. In the last decade, with the booming number of congenital and acquired metabolic disorders identified [71], the need to develop in vitro disease models via patient-derived hiPSCs to study pathogenic mechanisms and screen drugs preclinically is widely increasing. In the field of nucleotide sugar biosynthesis defects, to date only few hiPSC-based studies have been reported, specifically targeting phosphomannomutase 2 (PMM2) deficiency and UDP-glucose pyrophosphorylase (UGP2) deficiency [72,73,74], while they are more extensively used to model other inborn errors of metabolism, such as Pompe disease or mitochondrial disorders [75,76,77,78,79,80,81,82]. Nonetheless, despite the rapidly increasing interest in hiPSCs and hiPSC-derived models to study metabolic diseases, dynamic studies of metabolic pathways via isotopic labeling remains mostly unexplored. Therefore, the availability of chemically defined and customizable culture media would create new opportunities for applications of isotopic tracing in these models [17]. In this context, the customizable formulation of the FC-E8 medium (Supplementary Table S1) herein proposed enables the replacement of other nutrients beside glucose, such as labeled glutamine, asparagine and lipoic acid, to study reaction rates in nitrogen metabolism (using ^15^N-glutamine) or lipid metabolism (using for example ^13^C-labeled linoleic acid). Ultimately, the integration of metabolite level quantification with dynamic label-based studies provides a more complete overview of metabolic pathways and could help identify pathogenic mechanisms of diseases and potential targets for metabolic management.

## 5. Conclusions

In conclusion, in our study we successfully developed a chemically defined and customizable medium for stem cell culture suitable for ^13^C_6_-Glc labeling, and a UPHLC-MS/MS method for the detection of label incorporation in nucleotide sugars to deconvolute the contribution of different metabolic pathways to nucleotide sugar biosynthesis in pluripotent stem cells. By application of these tools, we provided the first example of isotope tracing of *de novo* nucleotide sugar biosynthesis in hiPSCs. Moreover, we presented the first translational application of our tools by performing dynamic isotopic labeling in hiPSCs derived from a patient affected by PGM1 deficiency, a metabolic disorder that affects the synthesis of UDP-glucose and UDP-galactose. Beyond PGM1 deficiency, the customizable formulation of FC-E8 medium is suitable for isotopic labeling not only via glucose but also via fatty acids and some amino acids, offering new opportunities for future applications in other metabolic pathways and metabolic disorders.

## Figures and Tables

**Table 1:** Phosphoglucomutase enzymatic activity assay on control and patient-derived hiPSCs.

hiPSC Line	PGM (mU/mg Protein)	PMI (mU/mg Protein)	Ratio PMI/PGM
hiPS-1	197.3	28.1	7.01
hiPS-2	185.3	18.8	9.85
hiPS-PGM1_1	5.2	28.9	0.18
hiPS-PGM1_2	6.5	28.1	0.23

## Data Availability

Additional data produced in this study are available upon request to the corresponding author.

## Supplementary Materials

The following supporting information can be downloaded at https://www.mdpi.com/article/10.3390/cells12131765/s1, Supplementary Figure S1: Characterization of PGM1-deficient patient-derived hiPSC lines. Supplementary Figure S2: Deconvolution of carbon fluxes contributing to the synthesis of the remaining 6 nucleotide sugars. Supplementary Figure S3: Incorporation of ^13^C atoms from glucose into nucleotide sugars mirrors the dynamics observed in hESCs but not in primary fibroblasts. Supplementary Figure S4: Results of isotopic tracing via ^13^C-glucose feeding of the remaining nucleotide sugars in control vs. PGM1-deficient hiPSCs. Supplementary Figure S5: Magnified brightfield pictures from Figure 2. Supplementary Figure S6: Magnified immunofluorescent pictures from Figure 2. Supplementary Figure S7: Unlabeled metabolomics analysis in control vs. PGM1-deficient hiPSCs. Supplementary Table S1: Composition of flux-conditioned E8 medium (light and heavy) compared to the composition of the custom DMEM-F12 and the E8 medium compositions. Supplementary Table S2: List of primers used for gene expression analysis of pluripotency markers. Supplementary Table S3: Commercial standards used for LC-MS method fine-tuning and as reference during the analysis. Supplementary Table S4: List of MRM transitions used for dynamic metabolic tracing analysis.
