# Supplementary material for: Isotopic Tracing of Nucleotide Sugar Metabolism in Human Pluripotent Stem Cells
Source: Cells. 2023 Jul 3;12(13):1765. doi: 10.3390/cells12131765 (PMC10340731; doi:10.3390/cells12131765)
Supplement: Supplementary file 1 [file cells-12-01765-s001.zip › cells-2259252-supplementary.pdf]

Isotopic tracing of nucleotide sugar metabolism in human pluripotent stem cells

Federica Conte, Marek J. Noga, Monique van Scherpenzeel, Raisa Veizaj, Rik Scharn, Juda-El Sam, Chiara Palumbo, Frans C.A. van den Brandt, Christian Freund, Eduardo Soares, Huiqing Zhou, Dirk J. Lefeber

Supplementary Figures and Tables

Supplementary Figure S1 2
Supplementary Figure S2 3
Supplementary Figure S3 4
Supplementary Figure S4 5
Supplementary Figure S5 6
Supplementary Figure S6 7
Supplementary Figure S7 8
Supplementary Table S1 9
Supplementary Table S2 11
Supplementary Table S3 11
Supplementary Table S4 12

Supplementary Figure S1. Characterization of PGM1-deficient patient-derived hiPSC lines. **(a)** Immunofluorescent staining of pluripotency markers SSEA-4, OCT-3/4 and Nanog in hiPS-PGM1_1 and hiPSC-PGM1_2. **(b)** Immunofluorescent staining of markers of spontaneous differentiation. AFP was used as marker for differentiation towards liver progenitors. CD31 was used as a marker for differentiation towards vessel endothelial cells. βIII-Tubulin was used as marker differentiation towards for neuronal progenitors.

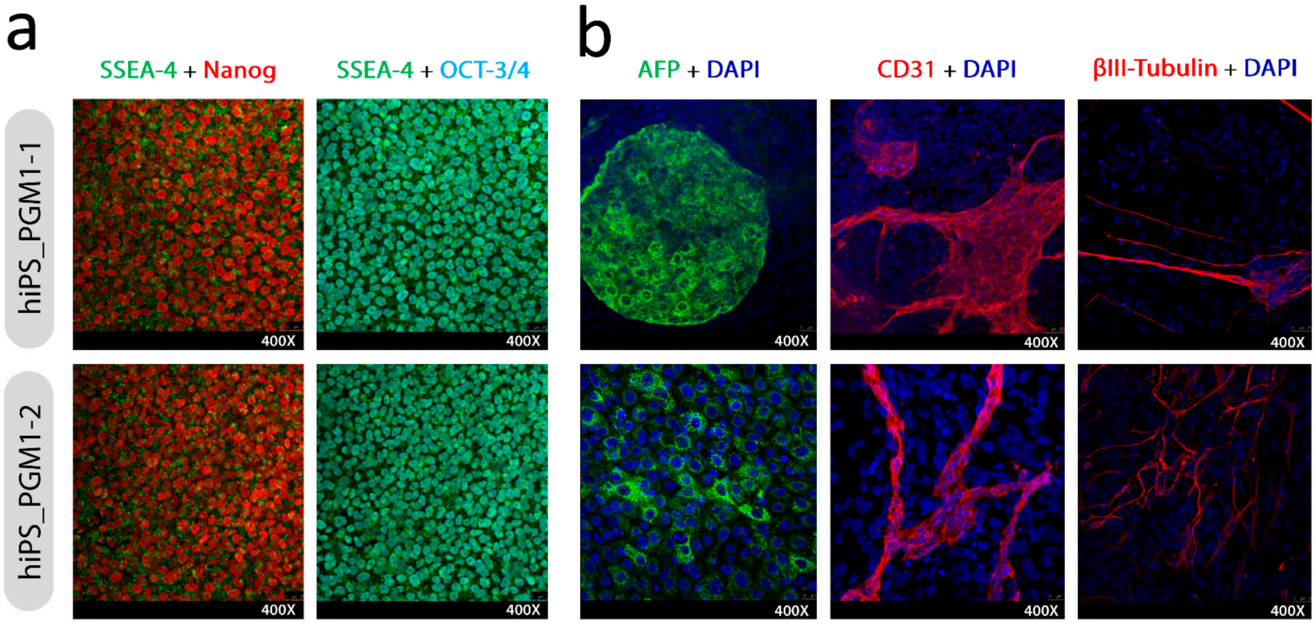

Supplementary Figure S2. Deconvolution of carbon fluxes contributing to the synthesis of the remaining 6 nucleotide sugars. **(a)** Schematic representation of the structures of the nucleotide sugars and products of their

fragmentation, including positions of labels derived from different metabolic precursors. **(b, c)** Time courses of isotopologues separated only based on their precursor mass **(b)**, and after label deconvolution based on MS/MS data **(c)**.

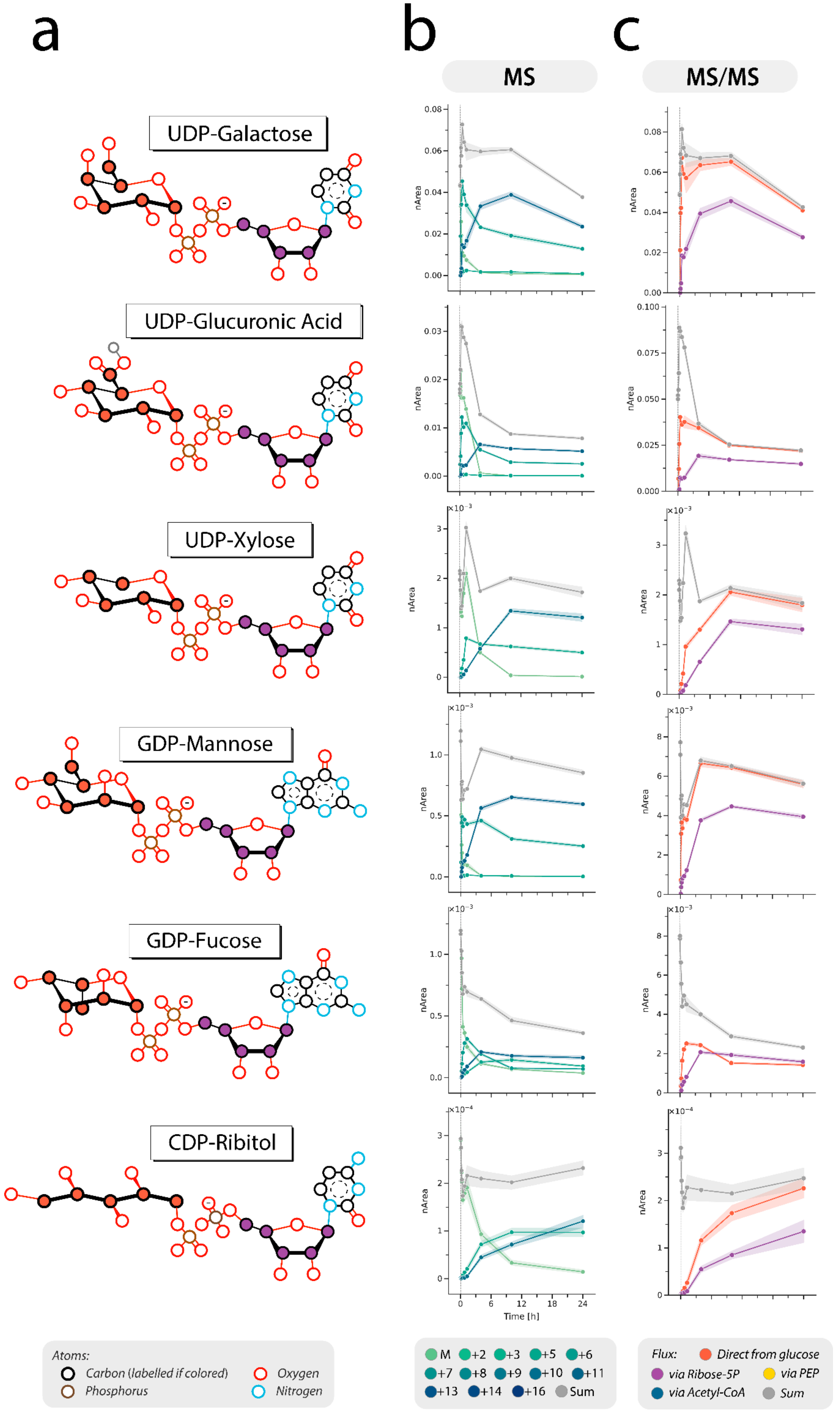

a
b
c
MS
MS/MS
UDP-Galactose
UDP-Glucuronic Acid
UDP-Xylose
GDP-Mannose
GDP-Fucose
CDP-Ribitol
nArea
Time [h]
Atoms:
Carbon (labelled if colored)
Oxygen
Phosphorus
Nitrogen
M
+2
+3
+5
+6
+7
+8
+9
+10
+11
+13
+14
+16
Sum
Flux:
Direct from glucose
via Ribose-5P
via PEP
via Acetyl-CoA
Sum

Supplementary Figure S3. Incorporation of ^{13}C atoms from glucose into nucleotide sugars mirrors the dynamics observed in hESCs but not in primary fibroblasts. Results of the isotopic labelling experiments indicating the time-course of incorporation of the labelled atoms in the six nucleotide sugars in two hiPSC lines (hiPS-1, hiPS-2), in one hESC line (hES), and in two primary dermal fibroblast lines (hFB-1, hFB2). Y-axis: normalized area (nArea) based on the total peak area method. X-axis: time expressed in hours (n=6).

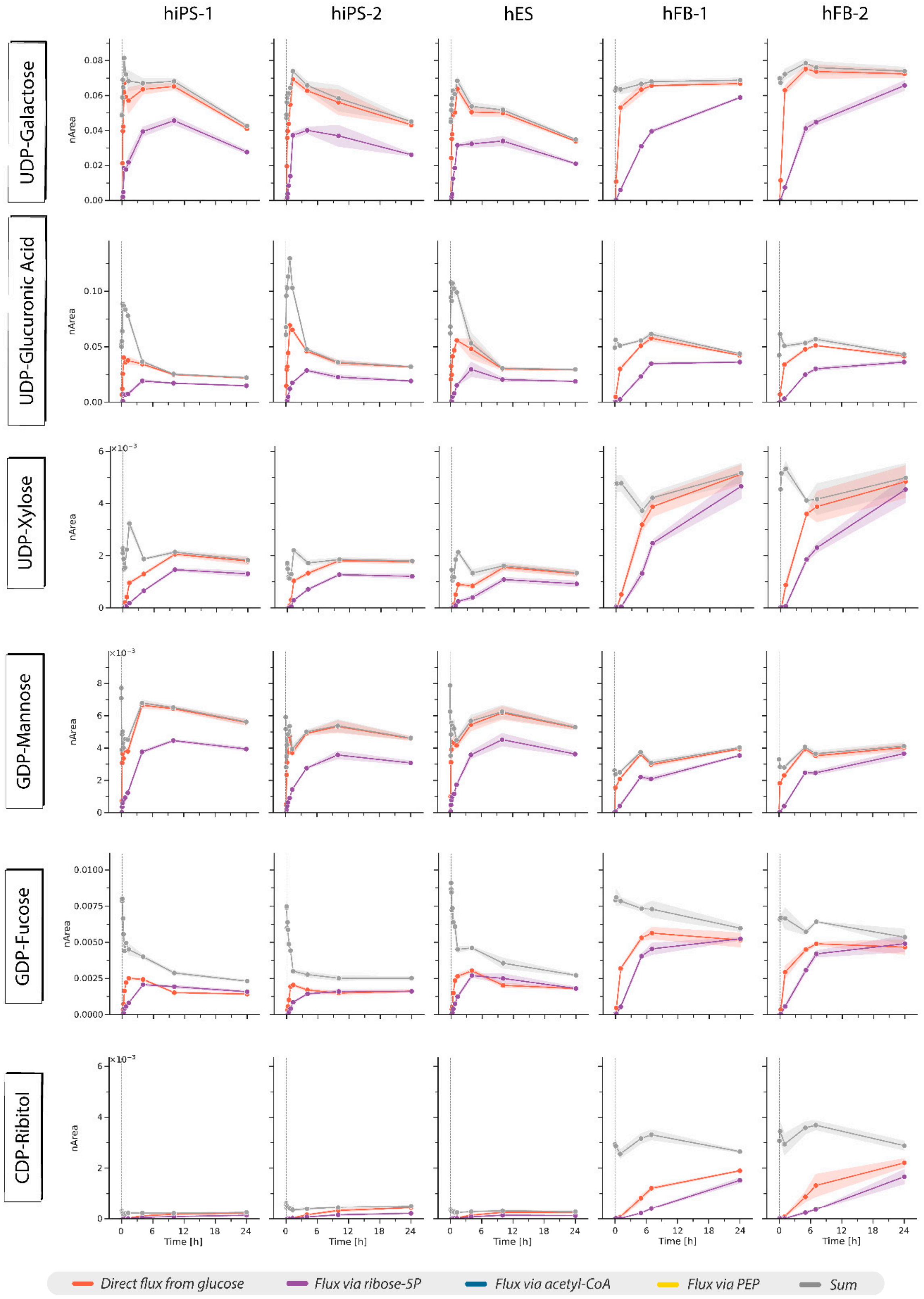
hiPS-1
hiPS-2
hES
hFB-1
hFB-2
UDP-Galactose
UDP-Glucuronic Acid
UDP-Xylose
GDP-Mannose
GDP-Fucose
CDP-Ribitol
nArea
Time [h]
Direct flux from glucose
Flux via ribose-5P
Flux via acetyl-CoA
Flux via PEP
Sum

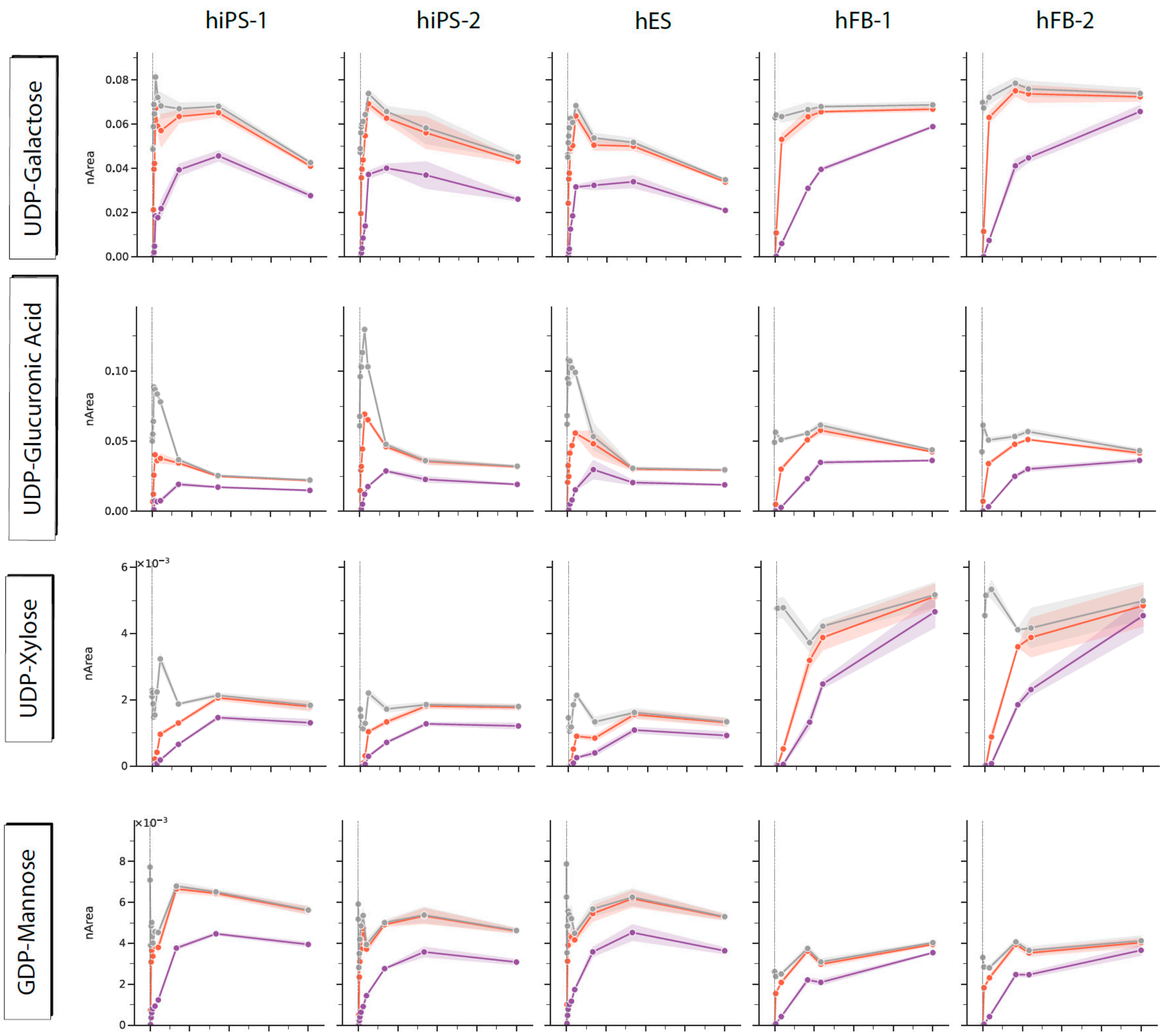

Supplementary Figure S4. Results of isotopic tracing via ^{13}C-glucose feeding of the remaining nucleotide sugars in control vs. PGM1-deficient hiPSCs. The plots indicate the time-course of incorporation of the labelled C atoms two hiPSC lines (hiPS-1, hiPS-2) and two PGM1-CDG patient-derived hiPSCs (hiPS-PGM1_1, hiPS-PGM1_2). Y-axis: normalized area (nArea) calculated using the total peak area method. X-axis: time expressed in hours (n=6).

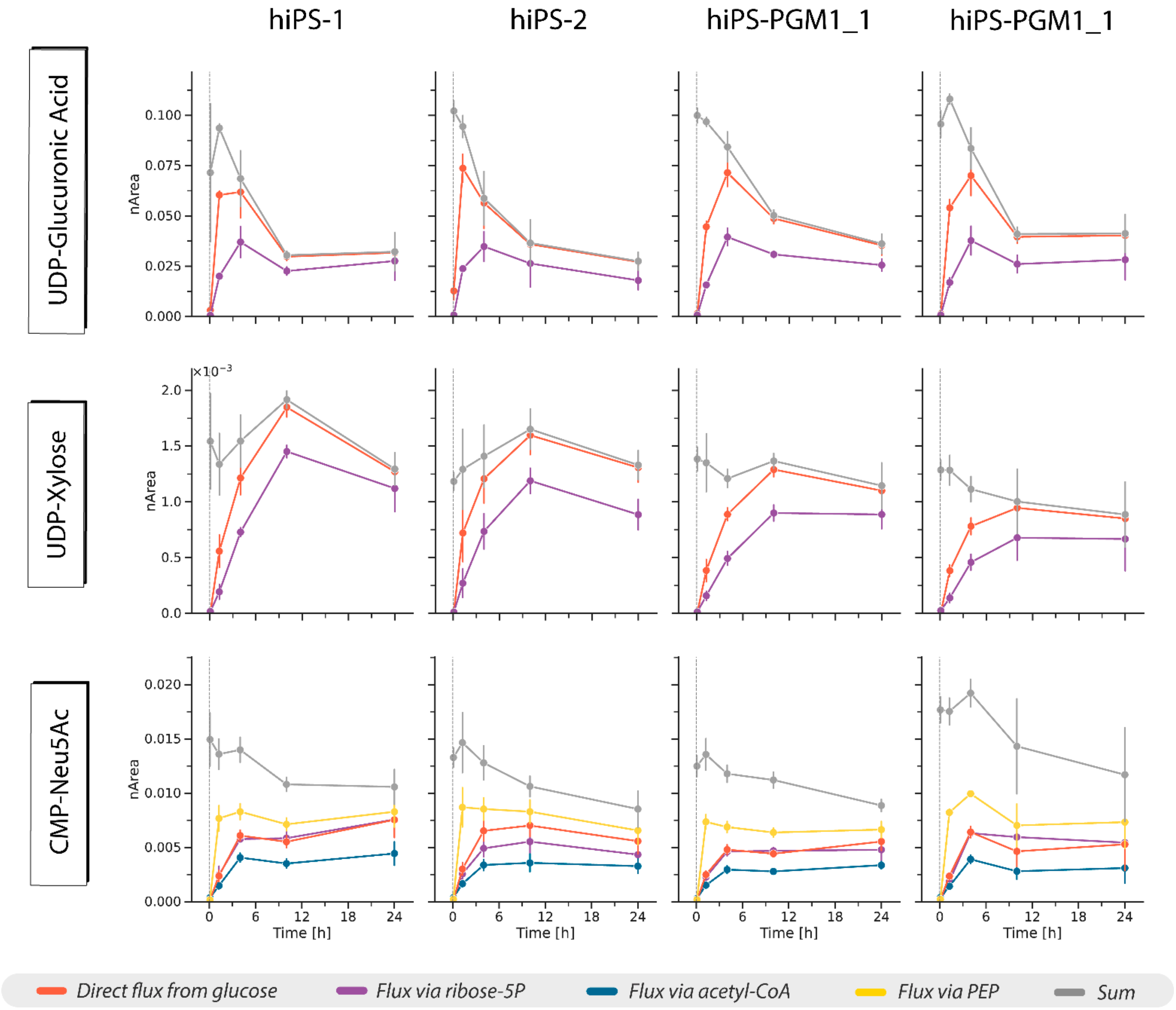

Supplementary Figure S5. Brightfield pictures of hiPS and hES cell lines cultured in FC-E8 for 24 hours. Magnified version of the brightfield picture reported in Figure 2. Scale bars: 500 µm.

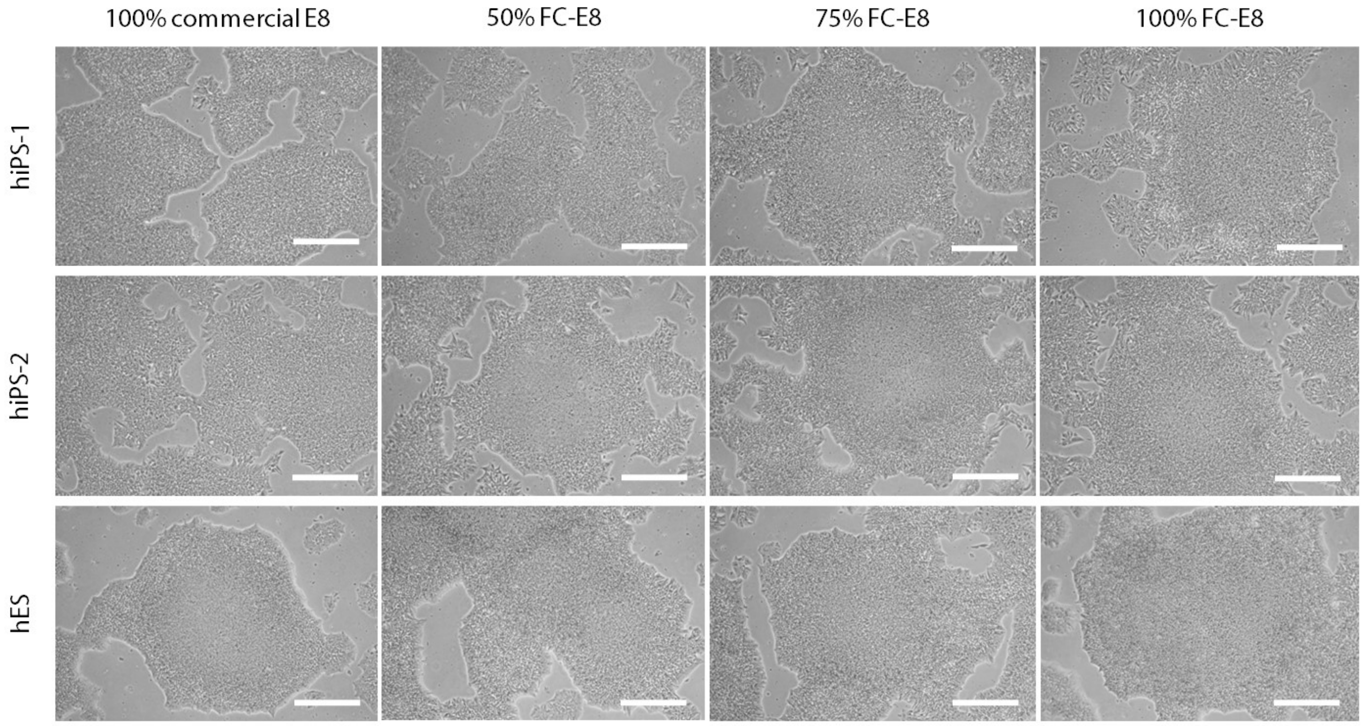

Supplementary Figure S6. Immunofluorescent pictures of hiPS and hES cell lines expanded and culture for additional four days in FC-E8. Magnified version of the immunofluorescent picture reported in Figure 2. Scale bars: 50 μm.

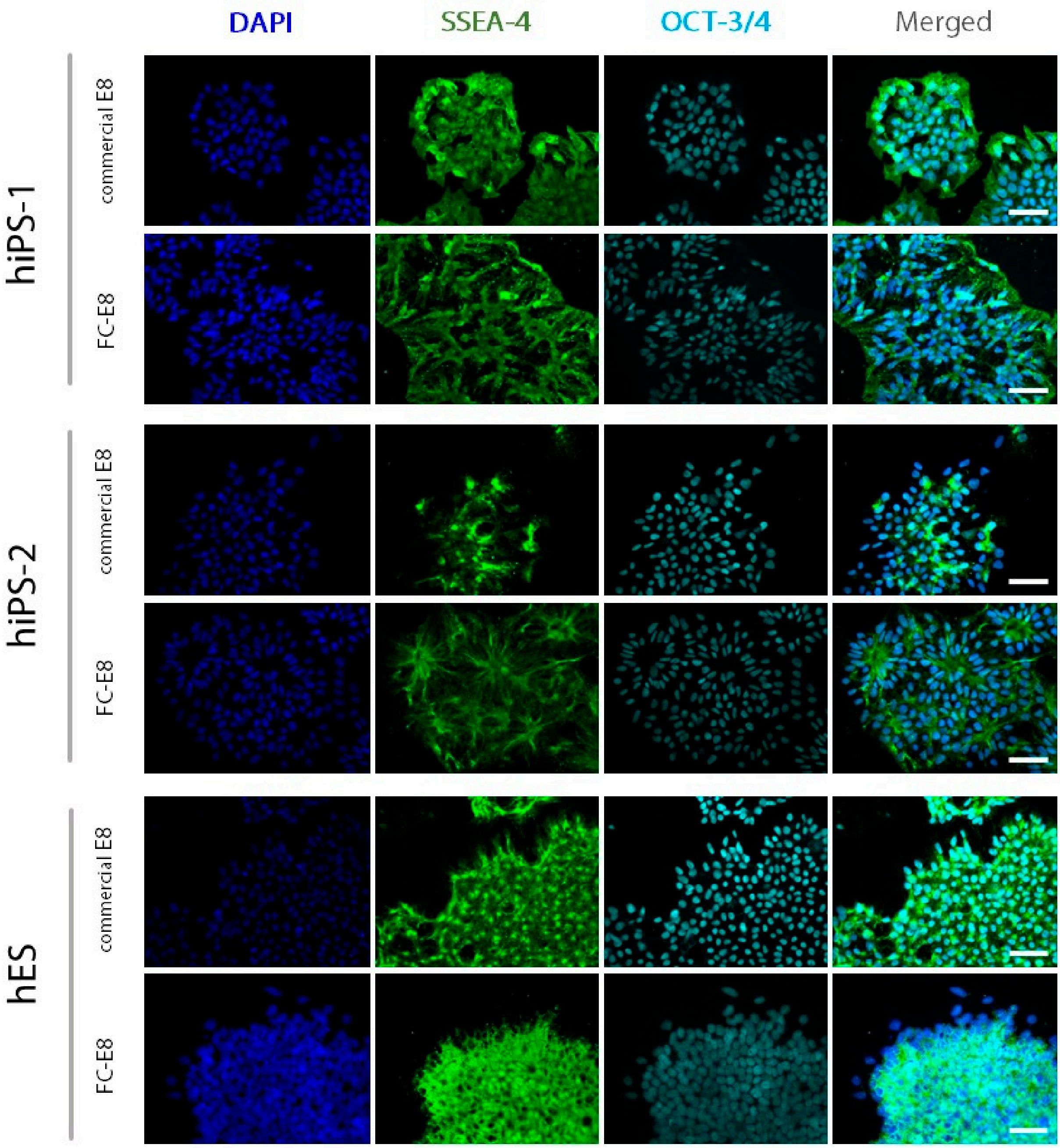

Supplementary Figure S7. Unlabeled metabolomics analysis in control vs. PGM1-deficient hiPSCs. Abbreviations: UDP-HexNAcs, UDP-*N*-acetyl hexosamine (UDP-GlcNAc + UDP-GalNAc); CMP-Neu5Ac, CMP-*N*-acetylneuraminic acid; UDP-, uridine 5'-diphosphate; CMP-, cytidine-5'-monophosphate; CDP-, cytidine-5'-

diphosphate; GDP-, Guanosine-5'-diphosphate. Y-axis: nucleotide sugar. X-axis: normalized area (nArea) based on total peak area method (n=6).

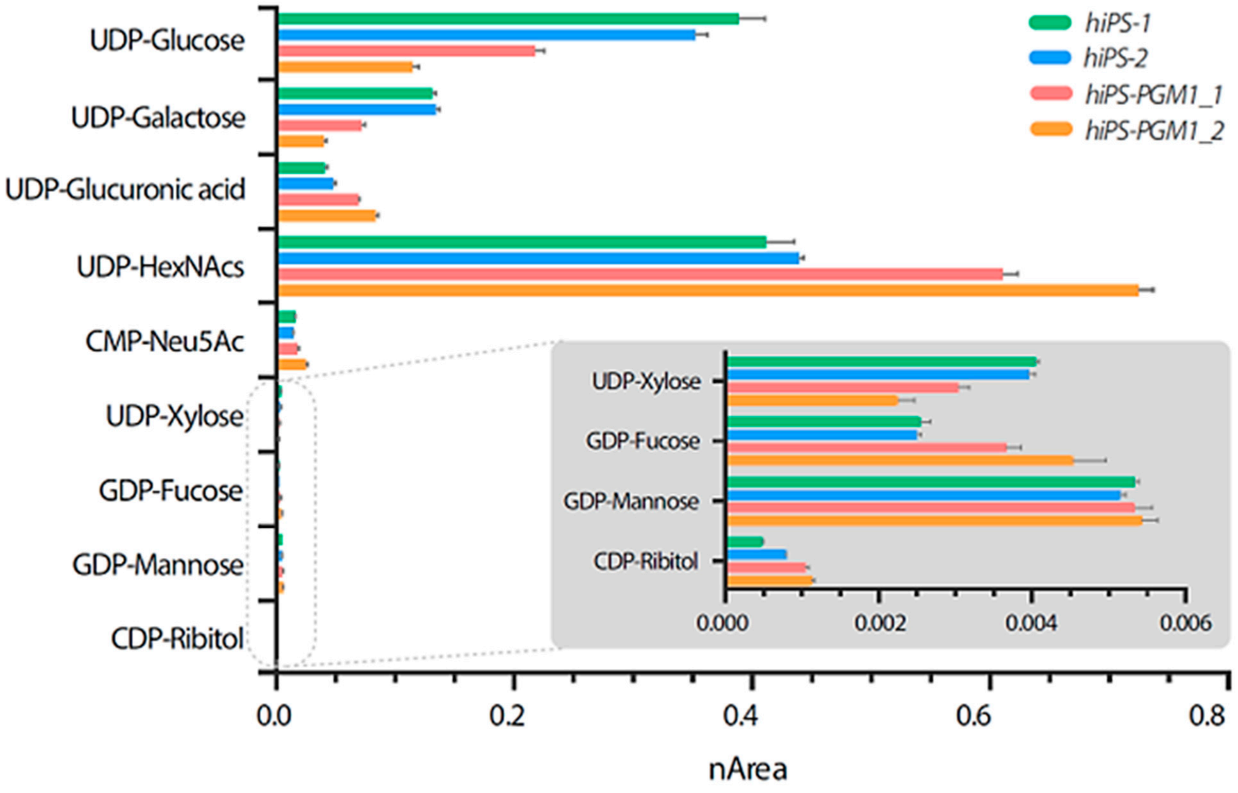

Supplementary Table S1. Composition of Flux-Conditioned E8 medium (light and heavy) compared to the composition of the custom DMEM-F12 and the E8 medium compositions.

Component	E8 according to Chen et al. 2011* (mg/L)	Custom DMEM/F-12 (mg/L)	Light FC-E8 (mg/L)	Heavy FC-E8 (mg/L)
Glycine	18.75	18.75	18.75	18.75
L-Alanine	4.45	4.45	4.45	4.45
L-Arginine hydrochloride	147.50	147.50	147.50	147.50
L-Asparagine	7.50	-	7.50	7.50
L-Aspartic acid	6.65	6.65	6.65	6.65
L-Cysteine hydrochloride	17.56	17.56	17.56	17.56
L-Cystine 2HCl	31.29	31.29	31.29	31.29
L-Glutamic acid	7.35	7.35	7.35	7.35
L-Glutamine	365.00	365.00	365.00	365.00
L-Histidine hydrochloride	31.48	31.48	31.48	31.48
L-Isoleucine	54.47	54.47	54.47	54.47
L-lysine hydrochloride	91.25	91.25	91.25	91.25
L-Methionine	17.24	17.24	17.24	17.24
L-Phenylalanine	35.48	35.48	35.48	35.48
L-Proline	17.25	17.25	17.25	17.25
L-Serine	26.25	26.25	26.25	26.25
L-Threonine	53.45	53.45	53.45	53.45
L-Tryptophan	9.02	9.02	9.02	9.02
L-Tyrosine disodium salt hydrate	55.79	55.79	55.79	55.79
L-Valine	52.85	52.85	52.85	52.85
Biotin	0.0035	0.0035	0.0035	0.0035
Choline chloride	8.98	8.98	8.98	8.98
D-Calcium pantothenate	2.24	2.24	2.24	2.24
Folic acid	2.65	2.65	2.65	2.65
Niacinamide	2.02	2.02	2.02	2.02
Pyridoxine hydrochloride	2.00	2.00	2.00	2.00
Riboflavin	0.219	0.219	0.219	0.219
Thiamine hydrochloride	2.17	2.17	2.17	2.17
Vitamin B12	0.68	0.68	0.68	0.68
Inositol	12.60	12.60	12.60	12.60
Calcium chloride (anhydrous)	116.60	116.60	116.60	116,60
Cupric sulfate	0.0013	0.0013	0.0013	0.0013
Ferric nitrate	0.05	0.05	0.05	0.05
Ferric sulfate	0.417	0.417	0.417	0.417
Magnesium chloride (anhydrous)	28.64	28.64	28.64	28.64

Magnesium sulfate (anhydrous)	48.84	48.84	48.84	48.84
Potassium chloride	311.80	311.80	311.80	311.80
Sodium chloride	6995.50	6995.50	6995.50	6995.50
Sodium phosphate dibasic anhydrous	71.02	71.02	71.02	71.02
Sodium phosphate monobasic	62.50	62.50	62.50	62.50
Zinc sulphate	0.432	0.432	0.432	0.432
Hypoxanthine Na	2.39	2.39	2.39	2.39
Linoleic Acid	0.042	-	0.042	0.042
Lipoic Acid	0.105	-	0.105	0.105
Petruscine 2HCl	0.081	0.081	0.081	0.081
Sodium Pyruvate	55.00	55.00	55.00	55.00
Thymidine	0.365	0.365	0.365	0.365
L-ascorbic acid-2-phosp. magnesium	64.00	-	64.00	64.00
Sodium selenite	14.00	-	14.00	14.00
Holo-Transferrin	10.70	-	10.70	10.70
Insulin	0.0194	-	0.0194	0.0194
FGF2	0.100	-	0.100	0.100
TGF-beta 1	0.002	-	0.002	0.002
Sodium bicarbonate	4181.00	2438.00	2981.00	2981.00
HEPES	3574.5	-	-	-
Glucose	3151.00	3151.00	3151.00	-
$^{13}C_6$-Glucose	-	-	-	3151.00

* *As the exact formulation of the commercial E8 medium (Gibco, Life Technologies) was not disclosed to public at the time of our study, we used as base the formulation of the E8 medium as published by Chen et al. 2011 [53].*

The compounds highlighted in orange color have been manually added in the medium during medium preparation.

Supplementary Table S2. List of primers used for gene expression analysis of pluripotency markers.

Gene symbol	Gene name (Ensembl gene ID)	Sequence primers	Product length
Nanog	Nanog homeobox (ENSG00000111704)	[Fw] AACCTCAGCTACAAACAGGTGA<br>[Rv] TCTGCGTCACACCATTGCTA	106 bp
CDH2	Cadherin 2 (ENSG00000170558)	[Fw] GAGGCTTCTGGTGAAATCGCA<br>[Rv] GCTTCTCACGGCATACACCA	207 bp
TERT	Telomerase reverse transcriptase (ENSG00000164362)	[Fw] AAAGCCAAGAACGCAGGGAT<br>[Rv] CGAGTCAGCTTGAGCAGGAA	110 bp
ACTG1	Actin gamma type 1 (ENSG00000184009)	[Fw] TTTCTCTGCCGGTCGCAAT<br>[Rv] CGATGGAAGGAAACACGGCT	120 bp

Supplementary Table S3. Commercial standards used for LC-MS method fine-tuning and as reference during the analysis.

Compound	*Manufacturer*	*Concentration in working solution*
UDP-α-D-glucose disodium salt	Sigma Aldrich (Merck)	10 μM
UDP-α-D-galactose disodium salt	Sigma Aldrich (Merck)	10 μM
UDP-glucuronic acid (UDP-GlcA) ammonium salt	Sigma Aldrich (Merck)	10 μM
UDP-Xylose	Carbosynth	10 μM
UDP-α-D-N-acetylglucosamine (UDP-GlcNAc) disodium salt	Sigma Aldrich (Merck)	10 μM
UDP-α-D-*N*-acetylgalactosamine (UDP-GalNAc) disodium salt	Sigma Aldrich (Merck)	10 μM
GDP-α-D-mannose disodium salt	Sigma Aldrich (Merck)	10 μM
GDP-β-L-fucose sodium salt	Sigma Aldrich (Merck)	10 μM
CMP-*N*-acetylneuraminic acid (CMP-Neu5Ac) sodium salt	Sigma Aldrich (Merck)	10 μM

Supplementary Table S4. List of MRM transitions used for dynamic metabolic tracing analysis.

Compound Name	Q1 mass (precursor ion) [amu]	Q3 mass (product ion) [amu]	Retention Time [min]	Delta Retention Time [min]	Collision Energy [eV]	Fragmentor	Cell Accelerator Voltage [V]	Polarity
CDP-ribitol	536.1	402.0	4.9	3.0	24.00	380	7	Negative
CDP-ribitol	536.1	384.0	4.9	3.0	26.00	380	7	Negative
CDP-ribitol	536.1	322.0	4.9	3.0	22.00	380	7	Negative
CDP-ribitol	536.1	273.0	4.9	3.0	35.00	380	7	Negative
CDP-ribitol	536.1	158.9	4.9	3.0	45.00	380	7	Negative
CDP-ribitol	536.1	97.0	4.9	3.0	51.00	380	7	Negative
CDP-ribitol	536.1	79.0	4.9	3.0	60.00	380	7	Negative
13C5-CDP-Ribitol	541.1	407.0	5.0	3.0	24.00	380	7	Negative
13C5-CDP-Ribitol	541.1	402.0	5.0	3.0	24.00	380	7	Negative
13C5-CDP-Ribitol	541.1	389.0	5.0	3.0	26.00	380	7	Negative
13C5-CDP-Ribitol	541.1	384.0	5.0	3.0	26.00	380	7	Negative
13C5-CDP-Ribitol	541.1	327.1	5.0	3.0	22.00	380	7	Negative
13C5-CDP-Ribitol	541.1	322.0	5.0	3.0	22.00	380	7	Negative
13C5-CDP-Ribitol	541.1	278.0	5.0	3.0	35.00	380	7	Negative
13C5-CDP-Ribitol	541.1	273.0	5.0	3.0	35.00	380	7	Negative
13C5-CDP-Ribitol	541.1	158.9	5.0	3.0	45.00	380	7	Negative
13C5-CDP-Ribitol	541.1	97.0	5.0	3.0	51.00	380	7	Negative
13C5-CDP-Ribitol	541.1	79.0	5.0	3.0	60.00	380	7	Negative
13C10-CDP-ribitol	546.1	407.0	5.0	3.0	24.00	380	7	Negative
13C10-CDP-ribitol	546.1	389.0	5.0	3.0	26.00	380	7	Negative
13C10-CDP-ribitol	546.1	327.1	5.0	3.0	22.00	380	7	Negative
13C10-CDP-ribitol	546.1	278.0	5.0	3.0	35.00	380	7	Negative
13C10-CDP-ribitol	546.1	158.9	5.0	3.0	45.00	380	7	Negative
13C10-CDP-ribitol	546.1	97.0	5.0	3.0	51.00	380	7	Negative
13C10-CDP-ribitol	546.1	79.0	5.0	3.0	60.00	380	7	Negative
CMP-N-acetyl-neuraminic acid	613.1	370.1	5.1	3.0	31.00	380	7	Negative
CMP-N-acetyl-neuraminic acid	613.1	322.0	5.1	3.0	22.00	380	7	Negative
CMP-N-acetyl-neuraminic acid	613.1	97.0	5.1	3.0	51.00	380	7	Negative
CMP-N-acetyl-neuraminic acid	613.1	79.0	5.1	3.0	60.00	380	7	Negative
13C2-CMP-N-acetyl-neuraminic acid	615.1	372.1	5.1	3.0	31.00	380	7	Negative
13C2-CMP-N-acetyl-neuraminic acid	615.1	322.0	5.1	3.0	22.00	380	7	Negative
13C2-CMP-N-acetyl-neuraminic acid	615.1	97.0	5.1	3.0	51.00	380	7	Negative
13C2-CMP-N-acetyl-neuraminic acid	615.1	79.0	5.1	3.0	60.00	380	7	Negative
13C3-CMP-N-acetyl-neuraminic acid	616.2	373.1	5.1	3.0	31.00	380	7	Negative
13C3-CMP-N-acetyl-neuraminic acid	616.2	322.0	5.1	3.0	22.00	380	7	Negative
13C3-CMP-N-acetyl-neuraminic acid	616.2	97.0	5.1	3.0	51.00	380	7	Negative
13C3-CMP-N-acetyl-neuraminic acid	616.2	79.0	5.1	3.0	60.00	380	7	Negative
13C5-CMP-N-acetyl-neuraminic acid	618.2	375.1	5.1	3.0	31.00	380	7	Negative
13C5-CMP-N-acetyl-neuraminic acid	618.2	370.1	5.1	3.0	31.00	380	7	Negative
13C5-CMP-N-acetyl-neuraminic acid	618.2	327.1	5.1	3.0	22.00	380	7	Negative
13C5-CMP-N-acetyl-neuraminic acid	618.2	322.0	5.1	3.0	22.00	380	7	Negative
13C5-CMP-N-acetyl-neuraminic acid	618.2	97.0	5.1	3.0	51.00	380	7	Negative
13C5-CMP-N-acetyl-neuraminic acid	618.2	79.0	5.1	3.0	60.00	380	7	Negative
13C6-CMP-N-acetyl-neuraminic acid	619.2	376.1	5.1	3.0	31.00	380	7	Negative
13C6-CMP-N-acetyl-neuraminic acid	619.2	322.0	5.1	3.0	22.00	380	7	Negative
13C6-CMP-N-acetyl-neuraminic acid	619.2	97.0	5.1	3.0	51.00	380	7	Negative
13C6-CMP-N-acetyl-neuraminic acid	619.2	79.0	5.1	3.0	60.00	380	7	Negative
13C7-CMP-N-acetyl-neuraminic acid	620.2	372.1	5.1	3.0	31.00	380	7	Negative
13C7-CMP-N-acetyl-neuraminic acid	620.2	327.1	5.1	3.0	22.00	380	7	Negative
13C7-CMP-N-acetyl-neuraminic acid	620.2	97.0	5.1	3.0	51.00	380	7	Negative
13C7-CMP-N-acetyl-neuraminic acid	620.2	79.0	5.1	3.0	60.00	380	7	Negative
13C8-CMP-N-acetyl-neuraminic acid	621.2	378.1	5.1	3.0	31.00	380	7	Negative
13C8-CMP-N-acetyl-neuraminic acid	621.2	373.1	5.1	3.0	31.00	380	7	Negative
13C8-CMP-N-acetyl-neuraminic acid	621.2	327.1	5.1	3.0	22.00	380	7	Negative
13C8-CMP-N-acetyl-neuraminic acid	621.2	322.0	5.1	3.0	22.00	380	7	Negative
13C8-CMP-N-acetyl-neuraminic acid	621.2	97.0	5.1	3.0	51.00	380	7	Negative
13C8-CMP-N-acetyl-neuraminic acid	621.2	79.0	5.1	3.0	60.00	380	7	Negative
13C9-CMP-N-acetyl-neuraminic acid	622.2	379.1	5.1	3.0	31.00	380	7	Negative
13C9-CMP-N-acetyl-neuraminic acid	622.2	322.0	5.1	3.0	22.00	380	7	Negative
13C9-CMP-N-acetyl-neuraminic acid	622.2	97.0	5.1	3.0	51.00	380	7	Negative
13C9-CMP-N-acetyl-neuraminic acid	622.2	79.0	5.1	3.0	60.00	380	7	Negative
13C10-CMP-N-acetyl-neuraminic acid	623.2	375.1	5.1	3.0	31.00	380	7	Negative
13C10-CMP-N-acetyl-neuraminic acid	623.2	327.1	5.1	3.0	22.00	380	7	Negative
13C10-CMP-N-acetyl-neuraminic acid	623.2	97.0	5.1	3.0	51.00	380	7	Negative
13C10-CMP-N-acetyl-neuraminic acid	623.2	79.0	5.1	3.0	60.00	380	7	Negative
13C11-CMP-N-acetyl-neuraminic acid	624.2	381.1	5.1	3.0	31.00	380	7	Negative
13C11-CMP-N-acetyl-neuraminic acid	624.2	376.1	5.1	3.0	31.00	380	7	Negative
13C11-CMP-N-acetyl-neuraminic acid	624.2	327.1	5.1	3.0	22.00	380	7	Negative
13C11-CMP-N-acetyl-neuraminic acid	624.2	322.0	5.1	3.0	22.00	380	7	Negative
13C11-CMP-N-acetyl-neuraminic acid	624.2	97.0	5.1	3.0	51.00	380	7	Negative
13C11-CMP-N-acetyl-neuraminic acid	624.2	79.0	5.1	3.0	60.00	380	7	Negative
13C13-CMP-N-acetyl-neuraminic acid	626.2	378.1	5.1	3.0	31.00	380	7	Negative
13C13-CMP-N-acetyl-neuraminic acid	626.2	327.1	5.1	3.0	22.00	380	7	Negative
13C13-CMP-N-acetyl-neuraminic acid	626.2	97.0	5.1	3.0	51.00	380	7	Negative
13C13-CMP-N-acetyl-neuraminic acid	626.2	79.0	5.1	3.0	60.00	380	7	Negative
13C14-CMP-N-acetyl-neuraminic acid	627.2	379.1	5.1	3.0	31.00	380	7	Negative
13C14-CMP-N-acetyl-neuraminic acid	627.2	327.1	5.1	3.0	22.00	380	7	Negative
13C14-CMP-N-acetyl-neuraminic acid	627.2	97.0	5.1	3.0	51.00	380	7	Negative
13C14-CMP-N-acetyl-neuraminic acid	627.2	79.0	5.1	3.0	60.00	380	7	Negative
13C16-CMP-N-acetyl-neuraminic acid	629.2	381.1	5.1	3.0	31.00	380	7	Negative
13C14-CMP-N-acetyl-neuraminic acid	627.2	97.0	5.1	3.0	51.00	380	7	Negative
13C14-CMP-N-acetyl-neuraminic acid	627.2	79.0	5.1	3.0	60.00	380	7	Negative
13C16-CMP-N-acetyl-neuraminic acid	629.2	381.1	5.1	3.0	31.00	380	7	Negative

GDP-fucose	588.1	442.0	16.3	3.0	24.00	380	7	Negative
GDP-fucose	588.1	424.0	16.3	3.0	26.00	380	7	Negative
GDP-fucose	588.1	362.1	16.3	3.0	22.00	380	7	Negative
GDP-fucose	588.1	225.0	16.3	3.0	31.00	380	7	Negative
13C5-GDP-fucose	593.1	447.0	16.3	3.0	24.00	380	7	Negative
13C5-GDP-fucose	593.1	429.0	16.3	3.0	26.00	380	7	Negative
13C5-GDP-fucose	593.1	367.1	16.3	3.0	22.00	380	7	Negative
13C5-GDP-fucose	593.1	225.0	16.3	3.0	31.00	380	7	Negative
13C6-GDP-fucose	594.1	442.0	16.3	3.0	24.00	380	7	Negative
13C6-GDP-fucose	594.1	424.0	16.3	3.0	26.00	380	7	Negative
13C6-GDP-fucose	594.1	362.1	16.3	3.0	22.00	380	7	Negative
13C6-GDP-fucose	594.1	231.0	16.3	3.0	31.00	380	7	Negative
13C11-GDP-fucose	599.1	447.0	16.3	3.0	24.00	380	7	Negative
13C11-GDP-fucose	599.1	429.0	16.3	3.0	26.00	380	7	Negative
13C11-GDP-fucose	599.1	367.1	16.3	3.0	22.00	380	7	Negative
13C11-GDP-fucose	599.1	231.0	16.3	3.0	31.00	380	7	Negative
GDP-mannose	604.1	442.0	12.8	4.7	24.67	380	7	Negative
GDP-mannose	604.1	424.0	12.8	4.7	28.33	380	7	Negative
GDP-mannose	604.1	362.1	12.8	4.7	24.00	380	7	Negative
GDP-mannose	604.1	273.0	12.8	4.7	39.33	380	7	Negative
GDP-mannose	604.1	241.0	12.8	4.7	33.67	380	7	Negative
GDP-mannose	604.1	158.9	12.8	4.7	46.00	380	7	Negative
GDP-mannose	604.1	97.0	12.8	4.7	51.00	380	7	Negative
GDP-mannose	604.1	79.0	12.8	4.7	63.33	380	7	Negative
13C5-GDP-mannose	609.1	447.0	12.5	4.7	25.00	380	7	Negative
13C5-GDP-mannose	609.1	429.0	12.5	4.7	29.50	380	7	Negative
13C5-GDP-mannose	609.1	367.1	12.5	4.7	25.00	380	7	Negative
13C5-GDP-mannose	609.1	278.0	12.5	4.7	41.50	380	7	Negative
13C5-GDP-mannose	609.1	241.0	12.5	4.7	35.00	380	7	Negative
13C5-GDP-mannose	609.1	158.9	12.5	4.7	46.50	380	7	Negative
13C5-GDP-mannose	609.1	97.0	12.5	4.7	51.00	380	7	Negative
13C5-GDP-mannose	609.1	79.0	12.5	4.7	65.00	380	7	Negative
13C11-GDP-mannose	615.1	447.0	12.4	4.7	25.00	380	7	Negative
13C11-GDP-mannose	615.1	429.0	12.4	4.7	29.50	380	7	Negative
13C11-GDP-mannose	615.1	367.1	12.4	4.7	25.00	380	7	Negative
13C11-GDP-mannose	615.1	278.0	12.4	4.7	41.50	380	7	Negative
13C11-GDP-mannose	615.1	247.0	12.4	4.7	35.00	380	7	Negative
13C11-GDP-mannose	615.1	158.9	12.4	4.7	46.50	380	7	Negative
13C11-GDP-mannose	615.1	158.9	12.4	4.7	46.50	380	7	Negative
13C11-GDP-mannose	615.1	97.0	12.4	4.7	51.00	380	7	Negative
13C11-GDP-mannose	615.1	79.0	12.4	4.7	65.00	380	7	Negative
UDP-galactose	565.0	403.0	6.3	4.7	24.00	380	7	Negative
UDP-galactose	565.0	385.0	6.3	4.7	26.00	380	7	Negative
UDP-galactose	565.0	323.0	6.3	4.7	22.00	380	7	Negative
UDP-galactose	565.0	273.0	6.3	4.7	36.00	380	7	Negative
UDP-galactose	565.0	241.0	6.3	4.7	30.67	380	7	Negative
UDP-galactose	565.0	158.9	6.3	4.7	45.33	380	7	Negative
UDP-galactose	565.0	97.0	6.3	4.7	51.00	380	7	Negative
UDP-galactose	565.0	79.0	6.3	4.7	61.33	380	7	Negative
13C5-UDP-galactose	570.1	408.0	6.3	4.7	24.00	380	7	Negative
13C5-UDP-galactose	570.1	390.0	6.3	4.7	26.00	380	7	Negative
13C5-UDP-galactose	570.1	328.0	6.3	4.7	22.00	380	7	Negative
13C5-UDP-galactose	570.1	278.0	6.3	4.7	36.00	380	7	Negative
13C5-UDP-galactose	570.1	241.0	6.3	4.7	30.67	380	7	Negative
13C5-UDP-galactose	570.1	158.9	6.3	4.7	45.33	380	7	Negative
13C5-UDP-galactose	570.1	97.0	6.3	4.7	51.00	380	7	Negative
13C5-UDP-galactose	570.1	79.0	6.3	4.7	61.33	380	7	Negative
13C6-UDP-galactose	571.1	403.0	6.3	4.7	24.00	380	7	Negative
13C6-UDP-galactose	571.1	385.0	6.3	4.7	26.00	380	7	Negative
13C6-UDP-galactose	571.1	323.0	6.3	4.7	22.00	380	7	Negative
13C6-UDP-galactose	571.1	273.0	6.3	4.7	36.00	380	7	Negative
13C6-UDP-galactose	571.1	247.0	6.3	4.7	30.67	380	7	Negative
13C6-UDP-galactose	571.1	158.9	6.3	4.7	45.33	380	7	Negative
13C6-UDP-galactose	571.1	97.0	6.3	4.7	51.00	380	7	Negative
13C6-UDP-galactose	571.1	79.0	6.3	4.7	61.33	380	7	Negative
13C11-UDP-galactose	576.1	408.0	6.3	4.7	24.00	380	7	Negative
13C11-UDP-galactose	576.1	390.0	6.3	4.7	26.00	380	7	Negative
13C11-UDP-galactose	576.1	328.0	6.3	4.7	22.00	380	7	Negative
13C11-UDP-galactose	576.1	278.0	6.3	4.7	36.00	380	7	Negative
13C11-UDP-galactose	576.1	247.0	6.3	4.7	30.67	380	7	Negative
13C11-UDP-galactose	576.1	158.9	6.3	4.7	45.33	380	7	Negative
13C11-UDP-galactose	576.1	97.0	6.3	4.7	51.00	380	7	Negative
13C11-UDP-galactose	576.1	79.0	6.3	4.7	61.33	380	7	Negative
UDP-glucose	565.0	403.0	7.3	4.7	24.00	380	7	Negative
UDP-glucose	565.0	385.0	7.3	4.7	26.00	380	7	Negative
UDP-glucose	565.0	385.0	7.3	4.7	26.00	380	7	Negative
UDP-glucose	565.0	323.0	7.3	4.7	22.00	380	7	Negative
UDP-glucose	565.0	273.0	7.3	4.7	36.00	380	7	Negative
UDP-glucose	565.0	241.0	7.3	4.7	30.67	380	7	Negative
UDP-glucose	565.0	158.9	7.3	4.7	45.33	380	7	Negative
UDP-glucose	565.0	97.0	7.3	4.7	51.00	380	7	Negative
UDP-glucose	565.0	79.0	7.3	4.7	61.33	380	7	Negative
13C5-UDP-glucose	570.1	408.0	7.3	4.7	24.00	380	7	Negative
13C5-UDP-glucose	570.1	390.0	7.3	4.7	26.00	380	7	Negative
13C5-UDP-glucose	570.1	328.0	7.3	4.7	22.00	380	7	Negative
13C5-UDP-glucose	570.1	278.0	7.3	4.7	36.00	380	7	Negative
13C5-UDP-glucose	570.1	241.0	7.3	4.7	30.67	380	7	Negative
13C5-UDP-glucose	570.1	158.9	7.3	4.7	45.33	380	7	Negative
13C5-UDP-glucose	570.1	97.0	7.3	4.7	51.00	380	7	Negative
13C5-UDP-glucose	570.1	79.0	7.3	4.7	61.33	380	7	Negative
13C6-UDP-glucose	571.1	403.0	7.3	4.7	24.00	380	7	Negative
13C6-UDP-glucose	571.1	385.0	7.3	4.7	26.00	380	7	Negative

13C6-UDP-glucose	571.1	323.0	7.3	4.7	22.00	380	7	Negative
13C6-UDP-glucose	571.1	273.0	7.3	4.7	36.00	380	7	Negative
13C6-UDP-glucose	571.1	247.0	7.3	4.7	30.67	380	7	Negative
13C6-UDP-glucose	571.1	158.9	7.3	4.7	45.33	380	7	Negative
13C6-UDP-glucose	571.1	97.0	7.3	4.7	51.00	380	7	Negative
13C6-UDP-glucose	571.1	79.0	7.3	4.7	61.33	380	7	Negative
13C11-UDP-glucose	576.1	408.0	7.3	4.7	24.00	380	7	Negative
13C11-UDP-glucose	576.1	390.0	7.3	4.7	26.00	380	7	Negative
13C11-UDP-glucose	576.1	328.0	7.3	4.7	22.00	380	7	Negative
13C11-UDP-glucose	576.1	278.0	7.3	4.7	36.00	380	7	Negative
13C11-UDP-glucose	576.1	247.0	7.3	4.7	30.67	380	7	Negative
13C11-UDP-glucose	576.1	158.9	7.3	4.7	45.33	380	7	Negative
13C11-UDP-glucose	576.1	97.0	7.3	4.7	51.00	380	7	Negative
13C11-UDP-glucose	576.1	79.0	7.3	4.7	61.33	380	7	Negative
UDP-glucuronic acid	579.0	403.0	15.0	3.0	24.00	380	7	Negative
UDP-glucuronic acid	579.0	385.0	15.0	3.0	28.00	380	7	Negative
UDP-glucuronic acid	579.0	323.0	15.0	3.0	21.00	380	7	Negative
UDP-glucuronic acid	579.0	273.0	15.0	3.0	41.00	380	7	Negative
UDP-glucuronic acid	579.0	255.0	15.0	3.0	23.00	380	7	Negative
UDP-glucuronic acid	579.0	158.9	15.0	3.0	44.00	380	7	Negative
UDP-glucuronic acid	579.0	97.0	15.0	3.0	51.00	380	7	Negative
UDP-glucuronic acid	579.0	79.0	15.0	3.0	80.00	380	7	Negative
13C5-UDP-glucuronic acid	584.0	408.0	15.0	3.0	24.00	380	7	Negative
13C5-UDP-glucuronic acid	584.0	390.0	15.0	3.0	28.00	380	7	Negative
13C5-UDP-glucuronic acid	584.0	328.0	15.0	3.0	21.00	380	7	Negative
13C5-UDP-glucuronic acid	584.0	278.0	15.0	3.0	41.00	380	7	Negative
13C5-UDP-glucuronic acid	584.0	255.0	15.0	3.0	23.00	380	7	Negative
13C5-UDP-glucuronic acid	584.0	158.9	15.0	3.0	44.00	380	7	Negative
13C5-UDP-glucuronic acid	584.0	97.0	15.0	3.0	51.00	380	7	Negative
13C5-UDP-glucuronic acid	584.0	79.0	15.0	3.0	80.00	380	7	Negative
13C6-UDP-glucuronic acid	585.0	403.0	15.0	3.0	24.00	380	7	Negative
13C6-UDP-glucuronic acid	585.0	385.0	15.0	3.0	28.00	380	7	Negative
13C6-UDP-glucuronic acid	585.0	323.0	15.0	3.0	21.00	380	7	Negative
13C6-UDP-glucuronic acid	585.0	273.0	15.0	3.0	41.00	380	7	Negative
13C6-UDP-glucuronic acid	585.0	261.0	15.0	3.0	23.00	380	7	Negative
13C6-UDP-glucuronic acid	585.0	158.9	15.0	3.0	44.00	380	7	Negative
13C6-UDP-glucuronic acid	585.0	97.0	15.0	3.0	51.00	380	7	Negative
13C6-UDP-glucuronic acid	585.0	79.0	15.0	3.0	80.00	380	7	Negative
13C11-UDP-glucuronic acid	590.1	408.0	14.3	3.0	24.00	380	7	Negative
13C11-UDP-glucuronic acid	590.1	390.0	14.3	3.0	28.00	380	7	Negative
13C11-UDP-glucuronic acid	590.1	328.0	14.3	3.0	21.00	380	7	Negative
13C11-UDP-glucuronic acid	590.1	278.0	14.3	3.0	41.00	380	7	Negative
13C11-UDP-glucuronic acid	590.1	261.0	14.3	3.0	23.00	380	7	Negative
13C11-UDP-glucuronic acid	590.1	158.9	14.3	3.0	44.00	380	7	Negative
13C11-UDP-glucuronic acid	590.1	97.0	14.3	3.0	51.00	380	7	Negative
13C11-UDP-glucuronic acid	590.1	79.0	14.3	3.0	80.00	380	7	Negative
UDP-N-acetyl-D-glucosamine / UDP-N-acetyl-galact	606.1	403.0	8.7	3.3	25.33	380	7	Negative
UDP-N-acetyl-D-glucosamine / UDP-N-acetyl-galact	606.1	385.0	8.7	3.3	27.00	380	7	Negative
UDP-N-acetyl-D-glucosamine / UDP-N-acetyl-galact	606.1	323.0	8.7	3.3	27.67	380	7	Negative
UDP-N-acetyl-D-glucosamine / UDP-N-acetyl-galact	606.1	282.0	8.7	3.3	31.67	380	7	Negative
UDP-N-acetyl-D-glucosamine / UDP-N-acetyl-galact	606.1	273.0	8.7	3.3	36.33	380	7	Negative
UDP-N-acetyl-D-glucosamine / UDP-N-acetyl-galact	606.1	158.9	8.7	3.3	50.33	380	7	Negative
UDP-N-acetyl-D-glucosamine / UDP-N-acetyl-galact	606.1	97.0	8.7	3.3	51.00	380	7	Negative
UDP-N-acetyl-D-glucosamine / UDP-N-acetyl-galact	606.1	79.0	8.7	3.3	72.67	380	7	Negative
13C2-UDP-N-acetyl-glucosamine / 13C2-UDP-N-ace	608.1	403.0	8.7	3.3	25.50	380	7	Negative
13C2-UDP-N-acetyl-glucosamine / 13C2-UDP-N-ace	608.1	385.0	8.7	3.3	27.00	380	7	Negative
13C2-UDP-N-acetyl-glucosamine / 13C2-UDP-N-ace	608.1	323.0	8.7	3.3	27.50	380	7	Negative
13C2-UDP-N-acetyl-glucosamine / 13C2-UDP-N-ace	608.1	284.0	8.7	3.3	31.50	380	7	Negative
13C2-UDP-N-acetyl-glucosamine / 13C2-UDP-N-ace	608.1	273.0	8.7	3.3	36.50	380	7	Negative
13C2-UDP-N-acetyl-glucosamine / 13C2-UDP-N-ace	608.1	158.9	8.7	3.3	49.00	380	7	Negative
13C2-UDP-N-acetyl-glucosamine / 13C2-UDP-N-ace	608.1	97.0	8.7	3.3	51.00	380	7	Negative
13C2-UDP-N-acetyl-glucosamine / 13C2-UDP-N-ace	608.1	79.0	8.7	3.3	72.00	380	7	Negative
13C5-UDP-N-acetyl-glucosamine / UDP-N-acetyl-ga	611.1	408.0	8.7	3.3	25.50	380	7	Negative
13C5-UDP-N-acetyl-glucosamine / UDP-N-acetyl-ga	611.1	390.0	8.7	3.3	27.00	380	7	Negative
13C5-UDP-N-acetyl-glucosamine / UDP-N-acetyl-ga	611.1	328.0	8.7	3.3	27.50	380	7	Negative
13C5-UDP-N-acetyl-glucosamine / UDP-N-acetyl-ga	611.1	282.0	8.7	3.3	31.50	380	7	Negative
13C5-UDP-N-acetyl-glucosamine / UDP-N-acetyl-ga	611.1	278.0	8.7	3.3	36.50	380	7	Negative
13C5-UDP-N-acetyl-glucosamine / UDP-N-acetyl-ga	611.1	158.9	8.7	3.3	49.00	380	7	Negative
13C5-UDP-N-acetyl-glucosamine / UDP-N-acetyl-ga	611.1	97.0	8.7	3.3	51.00	380	7	Negative
13C5-UDP-N-acetyl-glucosamine / UDP-N-acetyl-ga	611.1	79.0	8.7	3.3	72.00	380	7	Negative
13C6-UDP-N-acetyl-glucosamine / 13C6-UDP-N-ace	612.1	403.0	8.7	3.3	25.50	380	7	Negative
13C6-UDP-N-acetyl-glucosamine / 13C6-UDP-N-ace	612.1	385.0	8.7	3.3	27.00	380	7	Negative
13C6-UDP-N-acetyl-glucosamine / 13C6-UDP-N-ace	612.1	323.0	8.7	3.3	27.50	380	7	Negative
13C6-UDP-N-acetyl-glucosamine / 13C6-UDP-N-ace	612.1	288.1	8.7	3.3	31.50	380	7	Negative
13C6-UDP-N-acetyl-glucosamine / 13C6-UDP-N-ace	612.1	273.0	8.7	3.3	36.50	380	7	Negative
13C6-UDP-N-acetyl-glucosamine / 13C6-UDP-N-ace	612.1	158.9	8.7	3.3	49.00	380	7	Negative
13C6-UDP-N-acetyl-glucosamine / 13C6-UDP-N-ace	612.1	97.0	8.7	3.3	51.00	380	7	Negative
13C6-UDP-N-acetyl-glucosamine / 13C6-UDP-N-ace	612.1	79.0	8.7	3.3	72.00	380	7	Negative
13C7-UDP-N-acetyl-glucosamine / 13C7-UDP-N-ace	613.1	408.0	8.7	3.3	25.50	380	7	Negative
13C7-UDP-N-acetyl-glucosamine / 13C7-UDP-N-ace	613.1	390.0	8.7	3.3	27.00	380	7	Negative
13C7-UDP-N-acetyl-glucosamine / 13C7-UDP-N-ace	613.1	328.0	8.7	3.3	27.50	380	7	Negative
13C7-UDP-N-acetyl-glucosamine / 13C7-UDP-N-ace	613.1	284.0	8.7	3.3	31.50	380	7	Negative
13C7-UDP-N-acetyl-glucosamine / 13C7-UDP-N-ace	613.1	278.0	8.7	3.3	36.50	380	7	Negative
13C7-UDP-N-acetyl-glucosamine / 13C7-UDP-N-ace	613.1	158.9	8.7	3.3	49.00	380	7	Negative
13C7-UDP-N-acetyl-glucosamine / 13C7-UDP-N-ace	613.1	97.0	8.7	3.3	51.00	380	7	Negative
13C7-UDP-N-acetyl-glucosamine / 13C7-UDP-N-ace	613.1	79.0	8.7	3.3	72.00	380	7	Negative
13C8-UDP-N-acetyl-glucosamine / 13C8-UDP-N-ace	614.1	403.0	8.7	3.3	25.50	380	7	Negative
13C8-UDP-N-acetyl-glucosamine / 13C8-UDP-N-ace	614.1	385.0	8.7	3.3	27.00	380	7	Negative
13C8-UDP-N-acetyl-glucosamine / 13C8-UDP-N-ace	614.1	323.0	8.7	3.3	27.50	380	7	Negative
13C8-UDP-N-acetyl-glucosamine / 13C8-UDP-N-ace	614.1	290.1	8.7	3.3	31.50	380	7	Negative

13C8-UDP-N-acetyl-glucosamine / 13C8-UDP-N-ace	614.1	273.0	8.7	3.3	36.50	380	7	Negative
13C8-UDP-N-acetyl-glucosamine / 13C8-UDP-N-ace	614.1	158.9	8.7	3.3	49.00	380	7	Negative
13C8-UDP-N-acetyl-glucosamine / 13C8-UDP-N-ace	614.1	97.0	8.7	3.3	51.00	380	7	Negative
13C8-UDP-N-acetyl-glucosamine / 13C8-UDP-N-ace	614.1	79.0	8.7	3.3	72.00	380	7	Negative
13C11-UDP-N-acetyl-glucosamine / 13C11-UDP-N-a	617.1	408.0	8.7	3.3	25.50	380	7	Negative
13C11-UDP-N-acetyl-glucosamine / 13C11-UDP-N-a	617.1	390.0	8.7	3.3	27.00	380	7	Negative
13C11-UDP-N-acetyl-glucosamine / 13C11-UDP-N-a	617.1	328.0	8.7	3.3	27.50	380	7	Negative
13C11-UDP-N-acetyl-glucosamine / 13C11-UDP-N-a	617.1	288.1	8.7	3.3	31.50	380	7	Negative
13C11-UDP-N-acetyl-glucosamine / 13C11-UDP-N-a	617.1	278.0	8.7	3.3	36.50	380	7	Negative
13C11-UDP-N-acetyl-glucosamine / 13C11-UDP-N-a	617.1	158.9	8.7	3.3	49.00	380	7	Negative
13C11-UDP-N-acetyl-glucosamine / 13C11-UDP-N-a	617.1	97.0	8.7	3.3	51.00	380	7	Negative
13C11-UDP-N-acetyl-glucosamine / 13C11-UDP-N-a	617.1	79.0	8.7	3.3	72.00	380	7	Negative
13C13-UDP-N-acetyl-glucosamine / 13C13-UDP-N-a	619.1	408.0	8.7	3.3	25.50	380	7	Negative
13C13-UDP-N-acetyl-glucosamine / 13C13-UDP-N-a	619.1	390.0	8.7	3.3	27.00	380	7	Negative
13C13-UDP-N-acetyl-glucosamine / 13C13-UDP-N-a	619.1	328.0	8.7	3.3	27.50	380	7	Negative
13C13-UDP-N-acetyl-glucosamine / 13C13-UDP-N-a	619.1	290.1	8.7	3.3	31.50	380	7	Negative
13C13-UDP-N-acetyl-glucosamine / 13C13-UDP-N-a	619.1	278.0	8.7	3.3	36.50	380	7	Negative
13C13-UDP-N-acetyl-glucosamine / 13C13-UDP-N-a	619.1	158.9	8.7	3.3	49.00	380	7	Negative
13C13-UDP-N-acetyl-glucosamine / 13C13-UDP-N-a	619.1	97.0	8.7	3.3	51.00	380	7	Negative
13C13-UDP-N-acetyl-glucosamine / 13C13-UDP-N-a	619.1	79.0	8.7	3.3	72.00	380	7	Negative
UDP-xylose	535.0	403.0	7.2	5.2	23.00	380	7	Negative
UDP-xylose	535.0	385.0	7.2	5.2	25.50	380	7	Negative
UDP-xylose	535.0	323.0	7.2	5.2	21.00	380	7	Negative
UDP-xylose	535.0	273.0	7.2	5.2	34.50	380	7	Negative
UDP-xylose	535.0	211.0	7.2	5.2	32.00	380	7	Negative
UDP-xylose	535.0	158.9	7.2	5.2	44.00	380	7	Negative
UDP-xylose	535.0	97.0	7.2	5.2	51.00	380	7	Negative
UDP-xylose	535.0	79.0	7.2	5.2	65.00	380	7	Negative
13C5-UDP-xylose	540.1	408.0	7.3	5.2	23.00	380	7	Negative
13C5-UDP-xylose	540.1	403.0	7.3	5.2	23.00	380	7	Negative
13C5-UDP-xylose	540.1	390.0	7.3	5.2	25.50	380	7	Negative
13C5-UDP-xylose	540.1	385.0	7.3	5.2	25.50	380	7	Negative
13C5-UDP-xylose	540.1	328.0	7.3	5.2	21.00	380	7	Negative
13C5-UDP-xylose	540.1	323.0	7.3	5.2	21.00	380	7	Negative
13C5-UDP-xylose	540.1	278.0	7.3	5.2	34.50	380	7	Negative
13C5-UDP-xylose	540.1	273.0	7.3	5.2	34.50	380	7	Negative
13C5-UDP-xylose	540.1	216.0	7.3	5.2	32.00	380	7	Negative
13C5-UDP-xylose	540.1	211.0	7.3	5.2	32.00	380	7	Negative
13C5-UDP-xylose	540.1	158.9	7.3	5.2	44.00	380	7	Negative
13C5-UDP-xylose	540.1	97.0	7.3	5.2	51.00	380	7	Negative
13C5-UDP-xylose	540.1	79.0	7.3	5.2	65.00	380	7	Negative
13C10-UDP-xylose	545.1	408.0	7.2	5.2	23.00	380	7	Negative
13C10-UDP-xylose	545.1	390.0	7.2	5.2	25.50	380	7	Negative
13C10-UDP-xylose	545.1	328.0	7.2	5.2	21.00	380	7	Negative
13C10-UDP-xylose	545.1	278.0	7.2	5.2	34.50	380	7	Negative
13C10-UDP-xylose	545.1	216.0	7.2	5.2	32.00	380	7	Negative
13C10-UDP-xylose	545.1	158.9	7.2	5.2	44.00	380	7	Negative
13C10-UDP-xylose	545.1	97.0	7.2	5.2	51.00	380	7	Negative
13C10-UDP-xylose	545.1	79.0	7.2	5.2	65.00	380	7	Negative
